# Supplementary material for: AI-derived body composition parameters as prognostic factors in patients with HCC undergoing TACE in a multicenter study
Source: JHEP Rep. 2024 May 25;6(8):101125. doi: 10.1016/j.jhepr.2024.101125 (PMC11321290; doi:10.1016/j.jhepr.2024.101125)

AI-derived body composition parameters as prognostic factors in patients with HCC undergoing TACE in a multicenter study

Lukas Müller, Aline Mähringer-Kunz, Timo Alexander Auer, Uli Fehrenbach, Bernhard Gebauer, Johannes Haubold, Benedikt Michael Schaarschmidt, Moon-Sung Kim, René Hösch, Felix Nensa, Jens Kleesiek, Thierno D. Diallo, Michel Eisenblätter, Hanna Kuzior, Natascha Roehlen, Dominik Bettinger, Verena Steinle, Philipp Mayer, David Zopfs, Daniel Pinto Dos Santos, Roman Kloeckner

Table of contents

Table S1…………………………………………………………………………………2

Table S2…………………………………………………………………………………4

Table S3…………………………………………………………………………………6

Table S4…………………………………………………………………………………8

Table S5……………………………………………………………………………….10

Table S6……………………………………………………………………………….11

Table S7……………………………………………………………………………….12

Table S8……………………………………………………………………………….13

Table S9……………………………………………………………………………….14

Table S10………………………………………………………………………………15

Fig. S1…………………………………………………………………………………18

Fig. S2…………………………………………………………………………………19

Table S1. Baseline characteristics stratified by sex

**Variable**	**Male (*n* = 622)**	**Female (n=132)**	**p value**
Age in years, median (IQR)	67 (60–74)	67 (61 – 74)	0.682
Etiology of liver cirrhosis, *n* (%)			<0.001
Alcohol	283	29	
Viral	171	48	
Other	97	44	
Child-Pugh stage, *n* (%)			0.812
No cirrhosis	70	11	
A	322	71	
B	229	50	
BCLC stage, *n* (%)			0.263
0	8	2	
A	177	42	
B	316	72	
C	121	16	
Size of the largest lesion in mm, median (IQR)	38 (26 – 62)	39 (27 – 59)	0.967
Number of lesions, median (IQR)	1 (1 – 4)	2 (1 – 3)	0.627
Albumin level, g/L, median (IQR)	35 (30 – 39)	36 (30 – 39)	0.634

Bilirubin level, mg/dL, median (IQR)	1.1 (0.7 – 1.8)	1.3 (0.8 – 2.0)	0.105
Platelet count, per nL, median (IQR)	126 (85 – 190)	111 (77 – 180)	0.177
AST level, U/L, median (IQR)	59.0 (42.0 – 90.0)	68.5 (49.5 – 106.0)	0.007
ALT level, U/L, median (IQR)	40.0 (27.0 – 62.0)	41.0 (26.0 – 65.5)	0.662
INR, median (IQR)	1.2 (1.1 – 1.3)	1.2 (1.0 – 1.3)	0.494
AFP level, ng/mL, median (IQR)	21.7 (5.7 – 277.5)	44.0 (7.9 – 1015.0)	0.017

Comparison using Fisher test or Chi^2 for categorial variables and Mann-Whitney for continuous variables. P-values in bold show significant values.

Table S2. Baseline characteristics stratified by BCLC stage

**Variable**	**BCLC stage A (n=219)**	**BCLC stage B (n=388)**	**BCLC stage C (n=137)**	**p value**
Age in years, median (IQR)	65 (60 – 72)	68 (61 – 74)	66 (58 – 75)	0.033
Sex, *n* (%)				0.263
Female	42 (19.2)	72 (18.6)	16 (12.4)	
Male	177 (80.8)	316 (81.4)	121 (87.6)	
Etiology of liver cirrhosis, *n* (%)				0.047
Alcohol	100	162	48	
Viral	73	101	41	
Other	29	77	31	
Child-Pugh stage, *n* (%)				<0.001
No cirrhosis	17	48	17	
A	133	195	59	
B	69	145	61	
Size of the largest lesion in mm, median (IQR)	29 (23 – 39)	47 (33 – 65)	56 (33 – 87)	<0.001
Number of lesions, median (IQR)	1 (1 – 2)	3 (2 – 4)	2 (1 – 3)	<0.001
Albumin level, g/L, median (IQR)	36 (31 – 40)	35 (13 – 39)	32 (27 – 37)	<0.001
Bilirubin level, mg/dL, median (IQR)	1.1 (0.7 – 1.8)	1.2 (0.7 – 1.8)	1.2 (0.7 – 2.1)	0.812

Platelet count, per nL, median (IQR)	106 (77 – 165)	120 (84 – 194)	154 (105 – 234)	<0.001
AST level, U/L, median (IQR)	52.0 (38.0 – 70.0)	63.0 (44.3 – 94.8)	79.5 (51.3 – 114)	<0.001
ALT level, U/L, median (IQR)	38.0 (24.3 – 57.0)	41.0 (28.0 – 63.0)	41.0 (28.0 – 66.8)	0.266
INR, median (IQR)	1.2 (1.1 – 1.3)	1.1 (1.1 – 1.3)	1.2 (1.1 – 1.3)	0.242
AFP level, ng/mL, median (IQR)	10.4 (4.4 – 50.8)	33.6 (6.4 – 349.7)	148.0 (10.3 – 2639.7)	<0.001

Comparison using Fisher test or Chi^2 for categorial variables and Mann-Whitney for continuous variables. P-values in bold show significant values.

Table S3. BCA parameters stratified by sex

**Variable**	**Male (*n* = 622)**	**Female (n=132)**	**p value**
SM, mL	6160.1 (3468.6 – 7526.8)	4826.9 (2746.5 – 5900.4)	<0.001
TAT, mL	10904.8 (7636.3 – 15337.7	9640.0 (6965.0 – 15277.8)	0.231
IMAT, mL	922.3 (593.2 – 1493.5)	890.2 (523.3 – 1397.0)	0.284
SAT, mL	5495.2 (3573.3 – 8003.9)	6300.8 (4315.5 – 9942.4)	0.002
VAT, mL	4195.5 (2846.8 – 5878.4)	2400.1 (1667.4 – 3701.4)	<0.001
SM normalized*	74.1 (62.7 – 85.7)	59.6 (51.6 – 68.1)	<0.001
TAT normalized*	154.9 (112.3 – 197.3)	145.8 (106.8 – 189.3)	0.154
IMAT normalized*	12.8 (9.2 – 17.7)	11.3 (8.4 – 15.8)	0.121
SAT normalized*	74.2 (54.2 – 99.3)	92.3 (63.5 – 120.9)	<0.001
VAT normalized*	59.0 (39.3 – 80.6)	36.5 (23.4 – 50.7)	<0.001
SM/Bone	2.5 (2.2 – 2.8)	2.4 (2.1 – 2.7)	0.011
TAT/Bone	5.3 (3.8 – 6.9)	6.0 (4.2 – 7.6)	0.013
IMAT/Bone	0.4 (0.3 – 0.6)	0.5 (0.4 – 0.6)	0.068
SAT/Bone	2.6 (1.9 – 3.4)	3.8 (2.5 – 5.0)	<0.001

VAT/Bone	2.0 (1.3 – 2.8)	1.4 (1.0 – 2.1)	<0.001
Sarcopenia marker	1.7 (1.5 – 2.0)	1.6 (1.4 – 1.9)	0.002

Comparison using Mann-Whitney test. P-values in bold show significant values.

Table S4. BCA parameters stratified by BCLC stages

Variable	BCLC stage A	BCLC stage B	BCLC stage C	Overall p value
SM, mL	6018.9 (3978.4 – 7595.7)	5604.3 (3160.9 – 7359.5)	5510.7 (3352.3 – 7 241.2)	0.139
TAT, mL	11089.6 (7768.3 – 15844.3)	10986.1 (7618.2 – 15083.0)	10179.6 (6885.5 – 15068.0)	0.605
IMAT, mL	893.9 (620.5 – 1452.0)	944.6 (579.5 – 1456.4)	874.3 (557.6 – 1442.7)	0.819
SAT, mL	5942.8 (3988.1 – 8425.3)	5575.9 (3703.1 – 8265.3)	4981.3 (3496.5 – 7407.2)	0.219
VAT, mL	3685.0 (2409.7 – 5814.8)	3972.2 (2652.1 – 5404.4)	3913.6 (2444.6 – 5560.2)	0.950
SM normalized*	74.1 (61.1 – 87.5)	70.6 (59.0 – 82.5)	69.9 (60.6 – 81.8)	0.135
TAT normalized*	151.5 (106.7 – 204.3)	155.3 (113.8 – 190.7)	150.2 (114.4 – 190.0)	0.894
IMAT normalized*	12.1 (8.7 – 16.8)	13.0 (9.2 – 17.7)	12.4 (9.1 – 16.8)	0.710

SAT normalized*	76.7 (56.2 – 110.6)	78.1 (55.9 – 103.0)	72.7 (54.3 – 94.7)	0.460
VAT normalized*	55.4 (33.2 – 74.3)	54.1 (37.4 – 77.8)	56.2 (37.2 – 76.8)	0.722
SM/Bone	2.6 (2.2 – 2.9)	2.5 (2.2 – 2.8)	2.5 (2.2 – 2.8)	0.196
TAT/Bone	5.3 (3.7 – 7.2)	5.4 (4.0 – 7.1)	5.3 (3.8 – 6.7)	0.853
IMAT/Bone	0.43 (0.32 – 0.58)	0.45 (0.34 – 0.62)	0.45 (0.32 – 0.58)	0.462
SAT/Bone	2.7 (1.9 – 3.9)	2.7 (2.0 – 3.8)	2.5 (2.0 – 3.5)	0.568
VAT/Bone	1.8 (1.1 – 2.7)	1.9 (1.3 – 2.7)	2.0 (1.4 – 2.6)	0.570
Sarcopenia marker	1.8 (1.5 – 2.1)	1.7 (1.5 – 2.0)	1.7 (1.4 – 2.0)	0.187

All data are presented as median (IQR).

SM, skeletal muscle volume; TAT, total adipose tissue; IMAT, intermuscular adipose tissue; VAT, visceral adipose tissue; SAT, subcutaneous adipose tissue. *Volumes normalized to the slice number of the abdominal cavity. Kruskal-Wallis test for comparison. P-values in bold show significant values.

Table S5. Technical details of the performed TACE procedures

**Variable**	**All patients (*n* = 754)**
Type of TACE, *n* (%)	
cTACE	392 (52.0)
DEB-TACE	348 (46.2)
DSM-TACE	14 (1.8)
Level of selectivity, *n* (%)	
Selective	167 (22.1)
Superselective	587 (77.9)
Use of CBCT, *n* (%)	
Yes	69 (9.2)
No	685 (90.8)
Type of drug*, n	
Doxirubicin	390
Mitomycin	315
Epirubicin	277
Cisplatin	8
Irinotecan	1

cTACE, conventional transarterial chemoembolization; DEB-TACE, drug-eluting beads transarterial chemoembolization; DSM-TACE, degradable starch microspheres transarterial chemoembolization; CBCT, cone-beam computed tomography. *More than one drug possible.

Table S6. Correlation SM with other significant factors in multivariate analysis

**Variables**	**Correlation coefficient**	**Interpretation correlation**	**p value**
Albumin	0.168	Weak	**<0.001**
Bilirubin	-0.068	Negligible	0.062
AST	-0.030	Negligible	0.436
Max. lesion size	-0.075	Negligible	**0.047**

Interpretation: 10.1213/ANE.0000000000002864. Spearman correlation. P-values in bold show significant values.

Table S7. Univariate Cox regression analysis for additional clinical, laboratory and tumor burden-related variables apart from BCA parameters in all patients (continuous variables) (n = 754)

**Analysis**		**Univariate**		
**Covariate**		**HR**	**95% CI**	***p* value**
Age	Cont.	1.0	0.9–1.1	0.535
AFP	Cont.	1.1	1.0–1.1	**0.009**
Albumin	Cont.	0.6	0.5–0.7	**<0.001**
Bilirubin	Cont.	1.3	1.2–1.3	**<0.001**
AST level	Cont.	1.1	1.0–1.2	**<0.001**
ALT level	Cont.	1.0	0.9–1.1	0.370
INR level	Cont.	2.2	1.6–3.1	**<0.001**
Platelet count	Cont.	1.0	0.9–1.1	0.880
Tumor number	Cont.	1.2	1.1–1.3	**<0.001**
Max. lesion size	Cont.	1.1	1.0–1.2	**<0.001**

Cont., Continuous; AFP, alpha fetoprotein; AST, aspartate aminotransferase; ALT, alanine aminotransferase. P-values in bold show significant values.

Table S8. Univariate and multivariate Cox regression analysis for all after normalizing BCA parameter to patient height in patients with available height values (continuous variables) (n = 522)

**Analysis**		**Univariate**			**Multivariate**		
**Covariate**		**HR**	**95% CI**	***p* value**	**HR**	**95% CI**	***p* value**
Age	Cont.	1.0	0.9 – 1.1	0.485			
AFP	Cont.	1.1	1.0 – 1.2	**0.023**	1.0	1.0 – 1.1	0.432
Albumin	Cont.	0.6	0.6 – 0.7	**<0.001**	0.7	0.6 – 0.8	**<0.001**
Bilirubin	Cont.	1.3	1.1 – 1.4	**<0.001**	1.1	1.0 – 1.2	**0.009**
AST level	Cont.	1.1	1.0 – 1.2	**0.021**	1.2	0.9 – 1.4	0.192
ALT level	Cont.	1.0	0.8 – 1.1	0.690			
INR level	Cont.	1.8	1.1 – 2.8	**0.015**	0.8	0.4 – 1.5	0.443
Platelet count	Cont.	1.0	0.9 – 1.1	0.630			
Tumor number	Cont.	1.2	1.1 – 1.3	**<0.001**	1.1	1.0 – 1.3	**0.033**
Max. lesion size	Cont.	1.4	1.2 – 1.7	**<0.001**	1.3	1.2 – 1.6	**<0.001**
SM/height^2	Cont.	0.9	0.8 – 1.0	**0.018**	0.9	0.8 – 1.0	**0.047**
TAT/height^2	Cont.	0.9	0.8 – 1.0	**0.011**	1.0	0.8 – 1.1	0.520
IMAT/height^2	Cont.	0.9	0.8 – 1.0	0.190			
SAT/height^2	Cont.	0.9	0.8 – 1.0	**0.007**	0.9	0.8 – 1.0	0.187
VAT/height^2	Cont.	0.9	0.8 – 1.0	0.110			

Cont., Continuous; AFP, alpha fetoprotein; AST, aspartate aminotransferase; ALT, alanine aminotransferase; SM, skeletal muscle volume; TAT, total adipose tissue; IMAT, intermuscular adipose tissue; VAT, visceral adipose tissue; SAT, subcutaneous adipose tissue. *Volumes normalized to squared patient height. P-values in bold show significant values.

Table S9. Univariate Cox regression analysis for BCA parameters in all patients (continuous variables) stratified by sex

**Sex**		**Male (n=622)**			**Female (n=132)**		
**Covariate**		**HR**	**95% CI**	***p* value**	**HR**	**95% CI**	***p* value**
SM normalized	Cont.	0.9	0.8 – 1.0	**0.004**	0.8	0.6 – 1.0	**0.032**
TAT normalized	Cont.	0.9	0.8 – 1.0	**0.027**	0.9	0.7 – 1.1	0.213
IMAT normalized	Cont.	1.1	0.9 – 1.1	0.499	0.9	0.7 – 1.1	0.338
SAT normalized	Cont.	0.9	0.8 – 1.0	**0.023**	0.9	0.7 – 1.0	0.125
VAT normalized	Cont.	0.9	0.8 – 1.0	0.089	1.0	0.7 – 1.3	0.876
SM/Bone	Cont.	0.8	0.8 – 0.9	**<0.001**	0.9	0.8 – 1.0	**0.042**
TAT/Bone	Cont.	0.9	0.8 – 1.0	**0.029**	0.9	0.8 – 1.0	0.175
IMAT/Bone	Cont.	1.0	0.9 – 1.1	0.449	0.9	0.8 – 1.1	0.386
SAT/Bone	Cont.	0.9	0.8 – 1.0	**0.022**	0.9	0.8 – 1.1	0.280
VAT/Bone	Cont.	0.9	0.8 – 1.0	0.104	1.0	0.8 – 1.4	0.784
Sarcopenia marker	Cont.	0.9	0.8 – 1.0	**0.011**	0.9	0.8 – 1.0	0.124

SM, skeletal muscle volume; TAT, total adipose tissue; IMAT, intermuscular adipose tissue; VAT, visceral adipose tissue; SAT, subcutaneous adipose tissue. P-values in bold show significant values.

Table S10. Univariate Cox regression analysis for BCA parameters in all patients (continuous variables) stratified by BCLC stages (A, B, C)

**BCLC stage A (n=219)**				
**Covariate**		**HR**	**95% CI**	***p* value**
SM normalized	Cont.	0.9	0.8 – 1.0	0.087
TAT normalized	Cont.	1.1	0.9 – 1.3	0.434
IMAT normalized	Cont.	1.1	1.0 – 1.3	0.155
SAT normalized	Cont.	1.0	0.9 – 1.2	0.652
VAT normalized	Cont.	1.1	0.9 – 1.2	0.465
SM/Bone	Cont.	0.9	0.8 – 1.1	**0.043**
TAT/Bone	Cont.	1.0	0.9 – 1.2	0.599
IMAT/Bone	Cont.	1.1	0.9 – 1.3	0.209
SAT/Bone	Cont.	1.1	0.9 – 1.2	0.782
VAT/Bone	Cont.	1.0	0.9 – 1.2	0.644
Sarcopenia marker	Cont.	0.9	0.7 – 1.0	**0.041**
**BCLC Stage B (n=388)**				
**Covariate**		**HR**	**95% CI**	***p* value**
SM normalized	Cont.	0.9	0.8 – 1.0	**0.033**
TAT normalized	Cont.	0.8	0.7 – 0.9	**0.001**

IMAT normalized	Cont.	0.9	0.8 – 1.0	**0.032**
SAT normalized	Cont.	0.8	0.7 – 1.0	**0.008**
VAT normalized	Cont.	0.8	0.7 – 1.0	**0.007**
SM/Bone	Cont.	0.9	0.8 – 1.0	**0.027**
TAT/Bone	Cont.	0.8	0.7 – 1.0	**0.005**
IMAT/Bone	Cont.	0.9	0.8 – 1.0	**0.032**
SAT/Bone	Cont.	0.9	0.8 – 1.0	**0.021**
VAT/Bone	Cont.	0.9	0.8 – 1.0	**0.015**
Sarcopenia marker	Cont.	1.0	0.9 – 1.1	0.857
**BCLC Stage C (n=137)**				
**Covariate**		**HR**	**95% CI**	***p* value**
SM normalized	Cont.	0.8	0.7 – 1.0	**0.041**
TAT normalized	Cont.	0.9	0.7 – 1.1	0.155
IMAT normalized	Cont.	0.9	0.8 – 1.2	0.566
SAT normalized	Cont.	0.9	0.7 – 1.1	0.213
VAT normalized	Cont.	0.9	0.7 – 1.1	0.253
SM/Bone	Cont.	0.8	0.7 – 1.0	**0.039**

TAT/Bone	Cont.	0.9	0.7 – 1.1	0.168
IMAT/Bone	Cont.	0.9	0.7 – 1.2	0.540
SAT/Bone	Cont.	0.9	0.7 – 1.1	0.249
VAT/Bone	Cont.	0.9	0.8 – 1.1	0.285
Sarcopenia marker	Cont.	0.9	0.7 – 1.0	0.184

SM, skeletal muscle volume; TAT, total adipose tissue; IMAT, intermuscular adipose tissue; VAT, visceral adipose tissue; SAT, subcutaneous adipose tissue. P-values in bold show significant values.

Fig. S1. Relation between SM and OS with cubic regression spline (knots SM quantiles)

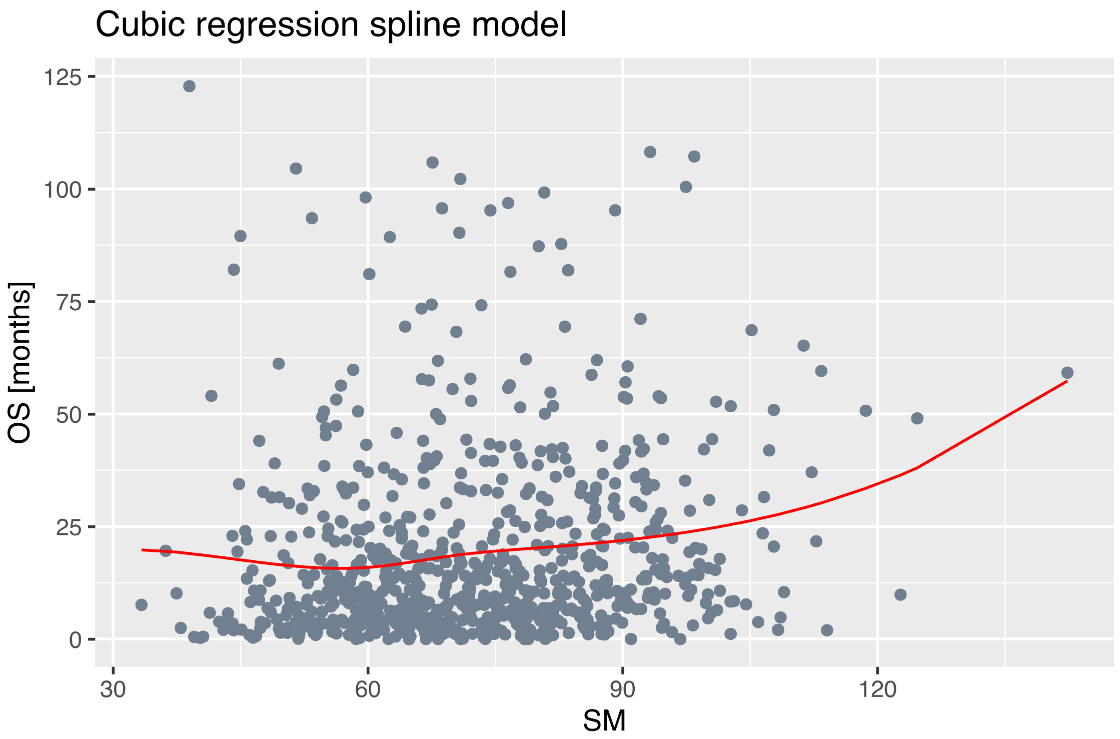

Fig. S2. Kaplan-Meier curves for the overall survival of all included patients, stratified according to previously published cut-off values for SM/Bone (Log-rank p=0.014) (A) and Sarcopenia Marker (Log-rank p<0.001) (B). SM, skeletal muscle volume.

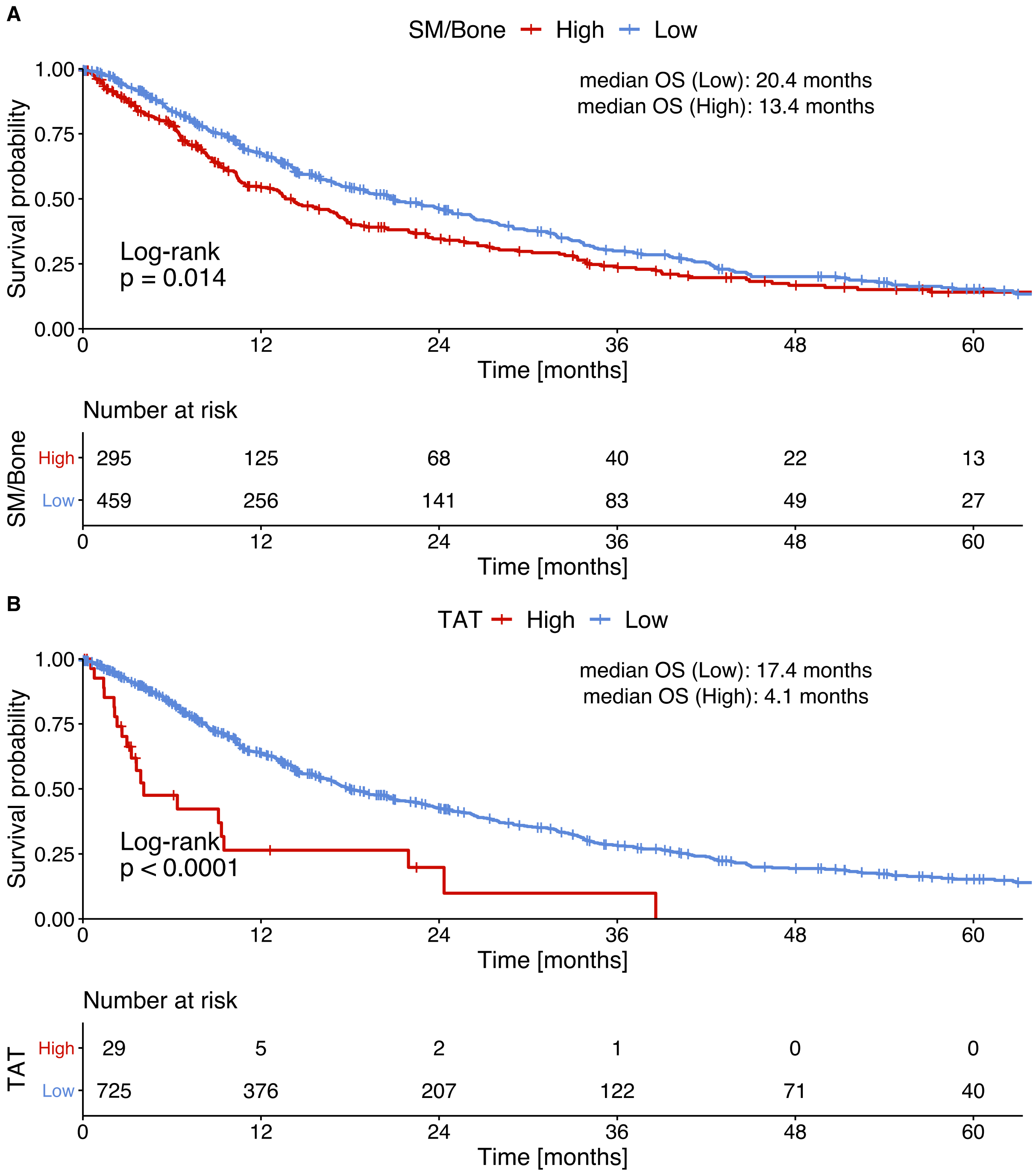

Supplement: Multimedia component 1 [file mmc1.pdf]
