# Supplementary material for: AI-derived body composition parameters as prognostic factors in patients with HCC undergoing TACE in a multicenter study
Source: JHEP Rep. 2024 May 25;6(8):101125. doi: 10.1016/j.jhepr.2024.101125 (PMC11321290; doi:10.1016/j.jhepr.2024.101125)

AI-derived body composition parameters as prognostic factors in patients with HCC undergoing TACE in a multicenter study

Lukas Müller[1], Aline Mähringer-Kunz[1], Timo Alexander Auer[2,3], Uli Fehrenbach[2], Bernhard Gebauer[2], Johannes Haubold[4,5], Benedikt Michael Schaarschmidt[4], Moon-Sung Kim[5], René Hosch[4,5], Felix Nensa[4,5], Jens Kleesiek[5], Thierno D. Diallo[6], Michel Eisenblätter[6,7], Hanna Kuzior[6], Natascha Roehlen[8], Dominik Bettinger[8], Verena Steinle[9], Philipp Mayer[9], David Zopfs[10], Daniel Pinto Dos Santos[10,11], Roman Kloeckner[12,*]

JHEP Reports **2024**. vol. 6 | 1–11

Check for updates

Background & Aims: Body composition assessment (BCA) parameters have recently been identified as relevant prognostic factors for patients with hepatocellular carcinoma (HCC). Herein, we aimed to investigate the role of BCA parameters for prognosis prediction in patients with HCC undergoing transarterial chemoembolization (TACE).

Methods: This retrospective multicenter study included a total of 754 treatment-naïve patients with HCC who underwent TACE at six tertiary care centers between 2010–2020. Fully automated artificial intelligence-based quantitative 3D volumetry of abdominal cavity tissue composition was performed to assess skeletal muscle volume (SM), total adipose tissue (TAT), intra- and intermuscular adipose tissue, visceral adipose tissue, and subcutaneous adipose tissue (SAT) on pre-intervention computed tomography scans. BCA parameters were normalized to the slice number of the abdominal cavity. We assessed the influence of BCA parameters on median overall survival and performed multivariate analysis including established estimates of survival.

Results: Univariate survival analysis revealed that impaired median overall survival was predicted by low SM (p <0.001), high TAT volume (p = 0.013), and high SAT volume (p = 0.006). In multivariate survival analysis, SM remained an independent prognostic factor (p = 0.039), while TAT and SAT volumes no longer showed predictive ability. This predictive role of SM was confirmed in a subgroup analysis of patients with BCLC stage B.

Conclusions: SM is an independent prognostic factor for survival prediction. Thus, the integration of SM into novel scoring systems could potentially improve survival prediction and clinical decision-making. Fully automated approaches are needed to foster the implementation of this imaging biomarker into daily routine.

© 2024 The Authors. Published by Elsevier B.V. on behalf of European Association for the Study of the Liver (EASL). This is an open access article under the CC BY license (http://creativecommons.org/licenses/by/4.0/).

Introduction

Hepatocellular carcinoma (HCC) is the most common primary liver malignancy, and among the leading causes of cancer-related deaths worldwide.[1] The European Association for the Study of the Liver (EASL) and the American Association for the Study of Liver Diseases (AASLD) both advocate use of the Barcelona Clinic Liver Cancer (BCLC) classification system as a basis for categorizing patients, allocating treatment, and predicting prognosis in individuals with HCC.[2,3] According to the current BCLC classification system, transarterial chemoembolization (TACE) is the treatment of choice for patients with intermediate-stage HCC.[4] However, in clinical reality, patients with intermediate-stage disease are a heterogenous group with remarkable variations in tumor burden and remaining liver function.[5] Additionally, in the concept of stage migration and individual treatment decision-making, TACE is also applied in other BCLC stages. Therefore, TACE is the most commonly applied treatment in patients with HCC,[6] and the patient heterogeneity makes it exceptionally difficult to perform risk scoring and prognosis prediction in patients treated with TACE.[7]

Novel approaches to risk scoring include the use of artificial intelligence (AI)-based automized risk calculation, and additional risk factors, apart from tumor burden and remaining liver function, are being investigated.[8] To achieve a more holistic picture of patients, and to have an objective indicator of patients' remaining capacities, body composition assessment (BCA) parameters have become vital as opportunistic imaging biomarkers in patients with hepatobiliary malignancies.[9–11]

Few studies have examined BCA parameters in patients with HCC undergoing TACE.[12–18] Moreover, most of these studies have had a monocentric design with a limited number of patients, and have been methodologically based on manual or semi-automatic measurement of BCA parameters, which

* Corresponding author. Address: Institute of Interventional Radiology, University Hospital of Schleswig-Holstein – Campus Lübeck, Ratzeburger Allee 160, 23538 Lübeck, Germany.
E-mail address: roman.kloeckner@uksh.de (R. Kloeckner).
https://doi.org/10.1016/j.jhepr.2024.101125

requires manual correction. This is a time-consuming task, which is not efficient for use in daily clinical routine. Furthermore, 2D-based methods provide only a rough estimation of tissue composition, and thus do not fulfill the requirements of state-of-the-art opportunistic computed tomography (CT) screening.[19] Furthermore, previous studies have typically relied on different surrogates for the body constitution, without considering the whole volume for the different tissue types in the abdominal cavity. This limits the comparability of the studies and their results. Additionally, prior studies have reported different results regarding the prognostic influence of skeletal muscle volume,[12–17] and the roles of different adipose tissues prior to treatment initiation have only been investigated in one single-center study to date.[20] Finally, no studies have been performed in European patient cohorts.

Novel AI-based algorithms enable fully automated, quantitative 3D volumetry of BCA parameters.[21] This could make it clinically realistic to integrate BCA parameters into interdisciplinary case discussions and treatment decision-making for patients with HCC undergoing TACE. However, there is an urgent need for multicentric large-scale evidence regarding the prognostic role of the different BCA parameters in these patients.

In the present multicentric study, we aimed to evaluate the roles of different BCA parameters for prognosis prediction in patients with HCC undergoing TACE. BCA parameters were assessed using a fully automated AI-based approach.

Patients and methods

This study protocol was approved by the Ethics Committee of the Medical Association of Rhineland-Palatinate, Germany (permit number 15913). The requirement for informed consent was waived due to the retrospective nature of the study. All other locally responsible ethics committees followed this decision. Patient records and information were anonymized at the local centers prior to data transfer. This report follows the guidelines for transparent reporting of a multivariable prediction model for individual prognosis or diagnosis.[22]

Patients

Six tertiary care centers in Germany participated in this study. The following inclusion criteria were applied: 1) first TACE between January 2010 and December 2020, to allow a minimum follow-up of 24 months; 2) age above 18 years; 3) histologically or image-based HCC diagnosis, according to the EASL criteria;[2] 4) no treatment performed prior to TACE; 5) no liver transplantation or tumor resection during the follow-up period after TACE; 6) BCLC stage within 0, A, B, or C; 7) pre-interventional abdominal CT scan available for BCA parameter calculation; and 8) full availability of clinical, laboratory, and imaging data. A total of 754 patients met all inclusion criteria and were included in the further analysis (Fig. 1).

Diagnosis, treatment, and data acquisition

HCC was diagnosed based on histological or image-derived EASL criteria, as previously reported.[2,23] Prior to their first TACE treatment, all patients underwent contrast-enhanced CT for diagnosis, staging, and treatment planning. Information on the imaging acquisition details have been previously reported.[24] TACE was performed in a standardized manner, as previously described in detail.[25–27] Follow-up comprised clinical examination, blood sampling, and cross-sectional imaging. Additionally, multidisciplinary tumor board discussion for further treatment decision-making was performed in case of HCC recurrence, tumor progression or change in either the patients' performance status or liver function. The primary endpoint of this study was median overall survival (OS), defined as the time interval between the initial TACE session and death or last follow-up. All baseline characteristics (including demographic data, liver disease status, and etiology), TACE-related parameters, and laboratory parameters were obtained from each hospital information system and from the laboratory database. Information regarding the tumor burden – including tumor growth pattern, number of lesions, and diameter of the largest target lesion – were determined based on the radiological report and the cross-sectional images. Radiologic response was assessed using mRECIST criteria.[2,28]

Body composition assessment

The BCA parameters were assessed using the solution for fully automated body composition in CT data previously published by Koitka *et al.*[21] This solution relies on a pre-trained convolutional neural network, and enables quantitative 3D volumetry of body tissue composition from standard abdominal CT examinations. In order to adapt the different acquisition slice thicknesses to each other, a resampling is carried out in advance by the algorithm itself to 5 mm slices. The algorithm was trained and validated on CT datasets with varying acquisition parameters. The following BCA parameters were analyzed: skeletal muscle volume (SM), total adipose tissue volume (TAT), intra- and intermuscular adipose tissue volume (IMAT), visceral adipose tissue volume (VAT), and subcutaneous adipose tissue volume (SAT). At each participating center, the data were analyzed using the Joint Imaging Platform of the German Cancer Consortium (DKTK) for federated data analysis and processing.[29] The abovementioned solution for BCA was transferred to the participating centers as a docker container. Thus, the medical image data remained at the different centers. The CT scans for each patient were sent from the local PACS (Picture Archiving and Communication System) to the Joint Imaging Platform server. Subsequently, data analysis using the algorithm was initiated. To counteract differences in patient size or scan range, the values of the body composition parameters SM, TAT, IMAT, SAT, and VAT volumes were normalized to the number of detected slices of the abdominal cavity (corresponding volume/number of detected slices), as previously published.[30] To further explore the prognostic role of the BCA parameters, the BCA parameter/bone ratio was examined as a prognostic factor as previously reported.[31,32] Furthermore, we calculated a sarcopenia marker (= muscle volume/(skeletal volume + IMAT)), which is also required for 3D BCA parameter analysis.[33] Furthermore, in a subset of patients with available body height (n = 522), we evaluated the BCA parameters/height^2 as previously reported.[31] In addition to evaluating these continuous factors, the study cohort was stratified using the available cut-offs for SM/bone ratio and BCA marker (Fig. S2).[31,33]

Statistical analysis

Statistical analyses and graphic design were performed in R 4.0.3 (A Language and Environment for Statistical Computing, R

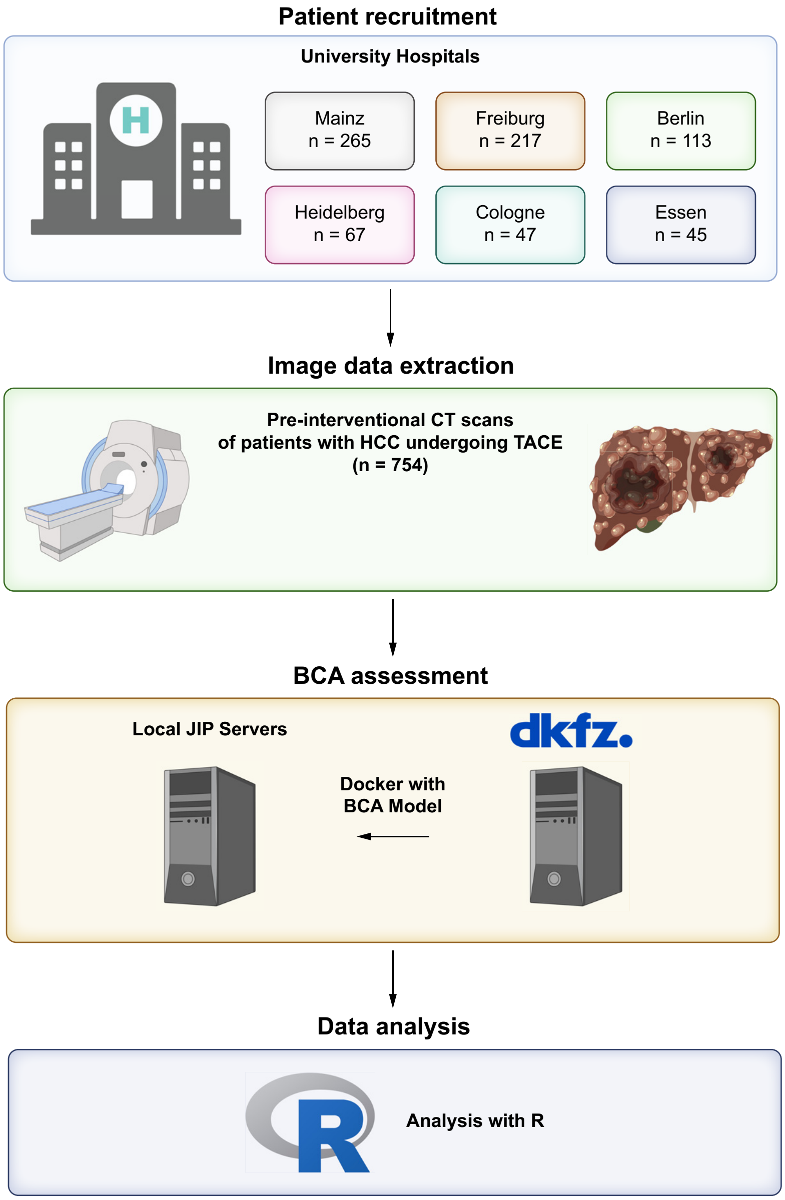

Fig. 1. Overview on the participating centers, the number of patients included, and the workflow for the BCA acquisition. BCA, body composition assessment; HCC, hepatocellular carcinoma; TACE, transarterial chemoembolization. Created with BioRender.com.

Foundation for Statistical Computing, http://www.R-project.org; last accessed July 16, 2023). Continuous data were reported as median and interquartile range, and categorical and binary baseline parameters as absolute number and percentage. Categorical parameters were compared using Fisher's exact test, and continuous parameters using the Mann-Whitney and Kruskal-Wallis test. Correlation coefficients were calculated according to Spearman. Univariate and multivariate Cox proportional hazards regression models were used to determine the effects of risk stratification, and to evaluate the roles of the analyzed factors. These findings were reported as hazard ratios (HRs) and corresponding 95% CIs. In a first step, Cox regression analysis was performed on unclassified continuous risk factors and the derived BCA parameters. To ensure interpretability and comparability of the HRs, continuous variables were subjected to Z-score normalization.[34] There are currently no standard values for 3D BCA in patients with liver diseases, including HCC. Thus, in a second step, we determined optimal BCA parameter cut-off values using optimal stratification methodology to identify clinically meaningful subgroups, with the packages "survminer" and "survival" (https://cran.r-project.org/package=survminer, https://CRAN.R-project.org/package=survival, accessed July 16, 2023). For this purpose, we used the "surv_cutpoint function", which determines the optimal cutpoint for a variable by using maximally selected rank statistics resulting in a cutpoint value that corresponds to the most significant relation with the outcome (https://www.rdocumentation.org/packages/survminer/versions/0.4.9/topics/surv_cutpoint). These packages were also used for the survival analyses. A p value of <0.05 was considered significant.

Results

Baseline characteristics

Of the 754 patients included, 82.5% were male, and the median age was 67 years. Table 1 presents the baseline characteristics at initial TACE treatment. In our study, more than 99% of the patients were Caucasian (n = 718), while the other patients were of Asian background (n = 6). The number of patients receiving multiple TACE sessions was n = 521 (69.1%). More detailed information on the performed TACE procedure can be found in Table S5. The median time between CT scan and TACE was 21 days (IQR 10–37 days). Baseline characteristics stratified according to sex and BCLC stages can be found in Tables S1 and S2.

Overview of BCA parameters in the cohort

Table 2 presents an overview of the BCA parameters in the cohort. BCA parameters stratified according to sex and BCLC stages can be found in Tables S3 and S4.

Influence of BCA parameters on survival after TACE

Median OS in the cohort was 17.4 months. Male patients had a slightly superior median OS compared to female patients (17.6 *vs.* 15.9 months, *p* = 0.760). In a first step, we assessed the prognostic ability of established risk factors, as well as BCA parameters, without dichotomization or any other form of categorization. In univariate survival analysis, independent prognostic factors included albumin, bilirubin, aspartate aminotransferase, international normalized ratio (INR), number of tumors, and maximum lesion size, as well as the BCA parameters SM, TAT, SAT, SM/bone, TAT/bone, Sarcopenia marker (Alatzides *et al.*), SM/height^2, TAT/height^2 (Table 3 and Table S7). When these parameters were included in multivariate analysis, among the included BCA parameters, SM, SM/bone and Sarcopenia marker remained independent prognostic factors (Table 4). We also performed a univariate and multivariate analysis in the subgroup of patients with available height and evaluated the role of BCA parameters in relation to height (as previously proposed[31]) (Table S8). Correlation of SM with other significant parameters in the multivariate analysis yielded a weak correlation (spearman's rho = 0.168) with albumin, while the correlation coefficients with the other parameters (bilirubin, aspartate aminotransferase, max. tumor size) were negligible (Table S6). Fig. S1 shows the relationship between SM and OS with a cubic regression spline with knots in the quantiles of SM.

In a second step, to form meaningful risk groups for translation into clinical application, we performed optimal cut-off fitting for the BCA parameters. Optimal cut-off fitting yielded the following values for the normalized BCA parameters: 89.0 for SM, 197.6 for TAT, 18.8 for IMAT, 119.5 for SAT, and 58.2 for VAT.

Compared to patients with a high SM volume, patients with a low SM volume exhibited a significantly impaired median OS (15.8 *vs.* 28.6 months, *p* <0.001, Fig. 2). Similarly, compared to patients with a low TAT volume, those with a high TAT volume had a significantly impaired median OS (15.3 *vs.* 32.1 months, *p* <0.001, Fig. 2). Additionally, compared to those with a low IMAT volume, patients with a high IMAT volume had an impaired

Table 1. Baseline characteristics.

Variable	All patients (N = 754)
Age in years, median (IQR)	67 (60–74)
Sex, n (%)	
Female	132 (17.5)
Male	622 (82.5)
No cirrhosis, n (%)	82 (10.9)
Etiology of cirrhosis, n (%)	
Alcohol	312 (41.4)
Viral	219 (29.0)
Other	141 (18.7)
Child-Pugh stage, n (%)	
No cirrhosis	82 (10.9)
A	393 (52.1)
B	279 (37.0)
BCLC stage, n (%)	
0	10 (1.3)
A	219 (29.0)
B	388 (51.5)
C	137 (18.2)
Portal vein invasion	
Yes	128 (17.0)
No	626 (83.0)
Extrahepatic Metastases	
Yes	44 (5.8)
No	710 (94.2)
Size of the largest lesion in mm, median (IQR)	39 (26 – 61)
Number of lesions, median (IQR)	2 (1–3)
Albumin level, g/L, median (IQR)	35 (30–39)
Bilirubin level, mg/dl, median (IQR)	1.2 (0.7–1.8)
Platelet count, per nl, median (IQR)	122 (84–187)
AST level, U/L, median (IQR)	61.0 (42.0–94.5)
ALT level, U/L, median (IQR)	40.0 (27.0–63.0)
INR, median (IQR)	1.2 (1.1–1.3)
AFP level, ng/ml, median (IQR)	24.0 (6.0–359.0)
Post-TACE treatment	
None	566 (75.1)
Systemic treatment	149 (19.8)
Locoregional treatment (SIRT, External radiation)	39 (5.2)

AFP, alpha fetoprotein; ALT, alanine aminotransferase; AST, aspartate aminotransferase; BCLC, Barcelona Clinic Liver Cancer; INR, international normalized ratio; TACE, transarterial chemoembolization.

Table 2. BCA parameters.

Variable	All patients (*n* = 754)
SM, ml	5,635.1 (3,387.1–7,412.5)
TAT, ml	10,795.1 (7,537.9–15,337.7)
IMAT, ml	903.3 (582.7–1,454.3)
SAT, ml	5,575.9 (3,692.1–8,259.7)
VAT, ml	3,911.8 (2,439.1–5,573.4)
SM normalized*	71.1 (60.1–83.4)
TAT normalized*	153.0 (110.2–195.9)
IMAT normalized*	12.6 (9.0–17.3)
SAT normalized*	76.7 (55.6–102.8)
VAT normalized*	54.2 (36.3–76.2)
SM/Bone	2.5 (2.2–2.8)
TAT/Bone	5.4 (3.8–7.1)
IMAT/Bone	0.5 (0.3–0.6)
SAT/Bone	2.7 (2.0–3.7)
VAT/Bone	1.9 (1.2–2.7)
Sarcopenia marker	2.5 (2.1–2.8)
SM/height^2**	23.6 (20.1–27.9)
TAT/height^2**	52.2 (38.1–66.9)
IMAT/height^2**	4.2 (3.1–5.9)
SAT/height^2**	26.2 (18.8–35.2)
VAT/height^2**	19.2 (12.8–26.6)

All data are presented as median (IQR).
BCA, body composition assessment; BCLC, Barcelona Clinic Liver Cancer; IMAT, intermuscular adipose tissue; SAT, subcutaneous adipose tissue; SM, skeletal muscle volume; TAT, total adipose tissue; VAT, visceral adipose tissue.
*Volumes normalized to the slice number of the abdominal cavity.
**Height only available in n = 522 patients.

Table 3. Univariate Cox regression analysis for the evaluated BCA parameters.

Covariate		HR	95% CI	*p* value
SM normalized*	Continuous	0.9	0.8–0.9	**<0.001**
TAT normalized*	Continuous	0.9	0.8–1.0	**0.013**
IMAT normalized*	Continuous	1.0	0.9–1.0	0.310
SAT normalized*	Continuous	0.9	0.8–1.0	**0.006**
VAT normalized*	Continuous	0.9	0.9–1.0	0.150
SM/Bone (MBR)	Continuous	0.7	0.6–0.8	**<0.001**
TAT/Bone	Continuous	0.9	0.9–1.0	**0.022**
IMAT/Bone	Continuous	0.1	0.0–92	0.300
SAT/Bone	Continuous	0.3	0.1–1.0	0.057
VAT/Bone	Continuous	0.4	0.1–2.3	0.280
Sarcopenia marker	Continuous	0.7	0.6–0.9	**<0.001**
SM/height^2**	Continuous	1.0	0.9–1.0	0.0118
TAT/height^2**	Continuous	1.0	1.0–1.0	**0.011**
IMAT/height^2**	Continuous	0.5	0.1–1.6	0.210
SAT/height^2**	Continuous	0.8	0.6–0.9	**0.009**
VAT/height^2**	Continuous	0.8	0.6–1.1	0.110

p values in bold show significant values.
BCA, body composition assessment; IMAT, intermuscular adipose tissue; SAT, subcutaneous adipose tissue; SM, skeletal muscle volume; TAT, total adipose tissue; VAT, visceral adipose tissue.
*Normalized on the slice thickness.
**Height only available in n = 522 patients.

median OS (16.5 *vs.* 27.2 months); however, this difference was not significant (*p* = 0.052, Fig. 2). Compared to patients with a low SAT volume, patients with a high SAT volume had a significantly impaired median OS (16.4 *vs.* 30.9 months, *p* <0.001 Fig. 2). Finally, compared to patients with a low VAT volume, those with a high VAT volume had a significantly impaired median OS (14.0 *vs.* 24.3 months, *p* = 0.030, Fig. 2).

Deeper analysis of sex-related and BCLC stage-related outcomes can be found in Tables S9 and S10.

In addition to the above-mentioned BCA parameters, albumin, bilirubin, INR, number of tumors, and maximum lesion size were independent prognostic factors in univariate survival analysis (Table 5). We performed multivariate analysis including all parameters that were independent prognostic factors in univariate survival analysis. Among the included BCA parameters, only SM remained an independent prognostic factor in multivariate analysis (*p* = 0.033, Table 5).

Subgroup analysis of patients within BCLC stage B

We performed subgroup analysis among 388 patients (51.5%) with BCLC stage B (*i.e.*, the subgroup for which TACE is recommended).[2,3] In univariate survival analysis, using the same cut-off values for the BCA parameters, the following were found to be significant prognostic factors: SM (*p* = 0.041), TAT (*p* <0.001), SAT (*p* = 0.015), and VAT (*p* = 0.001). Once again, IMAT was not a significant prognostic parameter (*p* = 0.150, Fig. 3). Univariate survival analysis also showed that albumin, bilirubin, and INR were independent prognostic factors (Table 6). Multivariate analysis was performed including the prognostic factors identified from univariate analysis. Among the BCA parameters, only SM remained an independent prognostic factor in multivariate analysis (HR 1.6, 95% CI 1.1–2.3, *p* = 0.012; Table 6).

Discussion

In this multicenter study, we identified skeletal muscle volume as an independent predictor of survival in patients with HCC undergoing TACE. This finding suggests that combining this valuable imaging biomarker with established markers could potentially improve patient stratification. Moreover, the automatized segmentation approach used in our study will make it feasible to analyze this marker by default, which may facilitate its clinical implementation.

CT datasets offer a wealth of image data that extends beyond the clinical indication for examination, and beyond the analytical capabilities of the radiologist's eye.[19] Research has been exploring advanced image analysis of tumor lesions in patients with HCC using radiomics or AI in-depth analysis, and expanding our knowledge of so-called opportunistic imaging biomarkers, such as body composition parameters.[35–38] Opportunistic CT screening "approaches incidental imaging data in a resourceful and systematic fashion by leveraging the wealth of body composition and other imaging findings available within all body CT scans".[19] This provides data about parameters that potentially have prognostic value far beyond that of tumor-associated characteristics, such as tumor size, number of tumor lesions, or growth patterns, as considered in current guidelines. However, as illustrated by the above quote from Perry Pickhardt, this requires systematic and resource-efficient analysis of the image data, not just the random detection of incidentalomas.[19] Previous studies of the prognostic relevance of BCA parameters in patients with HCC undergoing TACE have not met the necessary requirements for adequate opportunistic CT screening.[12–14,16,17,20] Notably, most of them have been based on manual recording of various surrogate parameters. Therefore, they are not systematic, in the sense that their reproducibility is clearly limited, nor do they meet the above-mentioned criteria regarding efficient use of available resources in daily radiological practice. Moreover, the previous studies have been limited to monocentric patient cohorts with mostly moderate numbers of patients, probably due to the high methodological effort required for manual extraction. In our present study, in addition to performing large-scale validation of the unclear data in patients with HCC undergoing TACE, our overarching goal was to demonstrate a highly automated approach that can be easily integrated into the daily radiological workflow. Importantly, our approach combines high reproducibility with a high degree of standardization, which greatly increases the methodological reliability. Additionally, we used a fully automated pipeline, which enabled direct transfer of the relevant scans from the local PACS to the AI platform. In this study, a freely available solution BCA algorithm was used that has been validated and used in previous studies.[31–33] Thus, the aim was to demonstrate the clinically relevant role that AI quantifications can play in the future as opportunistic image parameters for clinical decision-making in the use case presented. From a technical perspective, the present approach was to dissimilate a mature algorithm for BCA parameter assessment, using a Docker container system over a cross-center platform. This enabled easy multicenter distribution of algorithms with centralized data analysis, which is necessary for the required validations of BCA parameters in different cohorts, to generate evidence for routine clinical practice. Thus, our approach fulfills all of the above-mentioned criteria for opportunistic CT screening.

One major hindrance to clinical implementation of the BCA parameter is the current lack of standardized norm values. This is complicated by the shortage of large multicenter validation

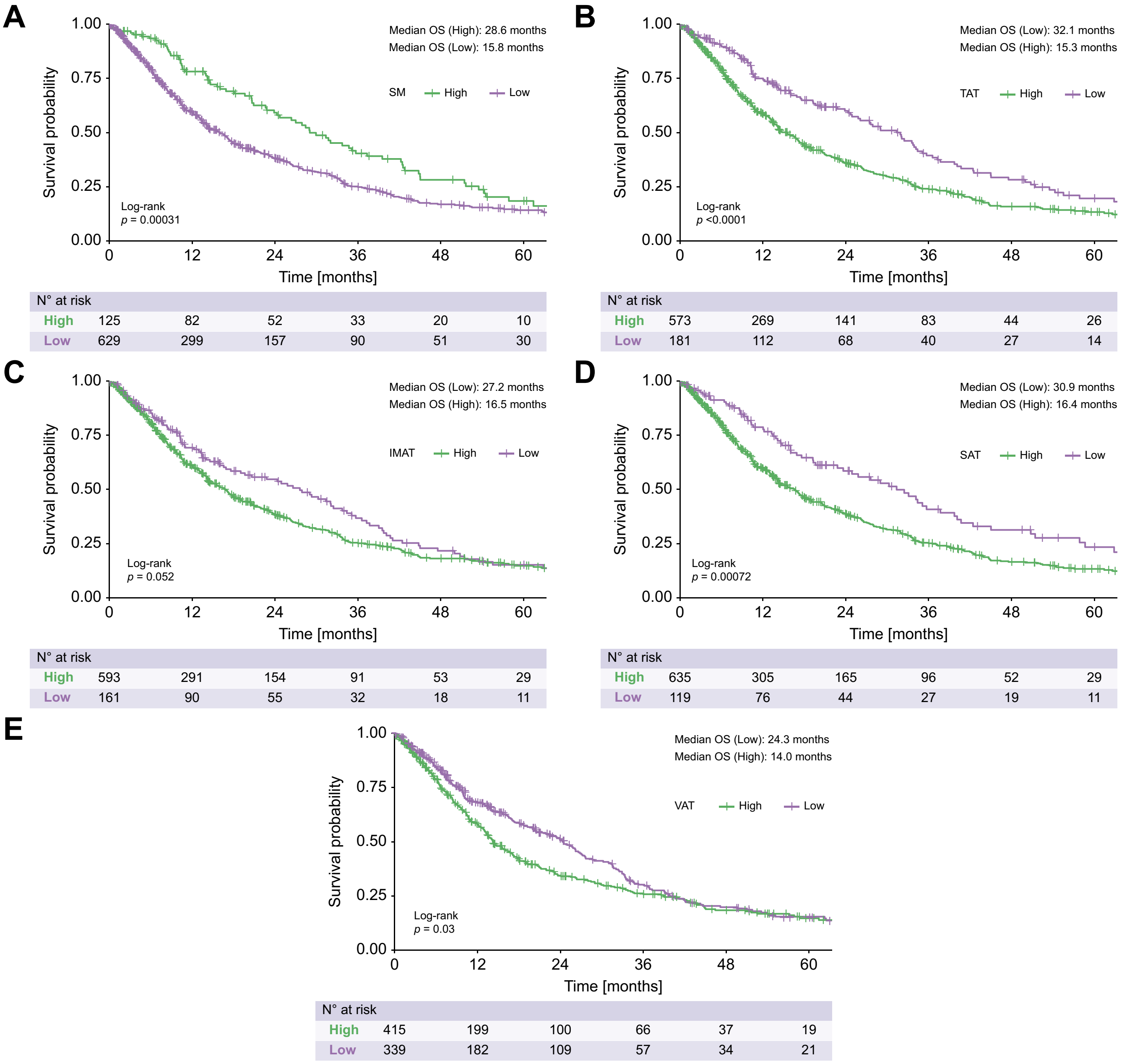

Fig. 2. Kaplan-Meier curves for the overall survival of all included patients, stratified according to their BCA parameters. All log rank test, *p* values: A <0.001, B <0.001, C = 0.052, D <0.001 E = 0.030). BCA, body composition assessment; IMAT, intermuscular adipose tissue; OS, overall survival; SAT, subcutaneous adipose tissue; SM, skeletal muscle volume; TAT, total adipose tissue; VAT, visceral adipose tissue.

studies for many patient groups. Therefore, one of our approaches was to provide standard values for 3D-based approaches, as we expect these to replace conventional 2D-based surrogate values in the future. At the same time, categorization naturally represents a source of error, sometimes leading to distortion of the valence of parameters.[39] To address this aspect, while still providing clinically applicable stratification values, we performed a two-stage evaluation in this study. In the first step, we compared the BCA parameters using a univariate and multivariate approach, without categorization with established risk factors. In the second step, we examined established optimal stratification values in our multicenter cohort. These values can be considered a benchmark for further evaluation and should be explored in larger clinical validation studies.

Regarding the role of muscle volume, we focused on assessment prior to treatment initiation. Our results showed that muscle volume was an independent prognostic factor, which is in line with previous studies that have investigated the role of parameters related to skeletal muscle volume prior to TACE initiation.[13–17] On the other hand, Fujita *et al.* compared the psoas muscle index before and after initiation of TACE in patients with HCC, in a longitudinal manner.[12] Interestingly, they found that the psoas muscle index prior to treatment initiation was not a relevant prognostic factor. Rather, only the psoas muscle index change after initiation of therapy was a

Table 4. Multivariate Cox regression analysis for all patients (continuous variables) (n = 754).

Covariate		Multivariate analysis HR	95% CI	*p* value
**BCA parameters**				
AFP	Continuous	1.0	1.0–1.1	0.347
Albumin	Continuous	0.6	0.5–0.7	**<0.001**
Bilirubin	Continuous	1.2	1.1–1.3	**<0.001**
AST level	Continuous	1.1	1.0–1.2	**0.003**
INR level	Continuous	1.0	0.9–1.1	0.524
Tumor number	Continuous	1.1	1.0–1.2	0.116
Max. lesion size	Continuous	1.3	1.1–1.5	**<0.001**
SM normalized*	Continuous	0.9	0.8–1.0	**0.039**
TAT normalized*	Continuous	1.0	1.0–1.0	0.424
SAT normalized*	Continuous	1.0	1.0–1.0	0.349
**BCA parameters/Bone**				
AFP	Continuous	1.0	1.0–1.1	0.306
Albumin	Continuous	0.6	0.5–0.7	**<0.001**
Bilirubin	Continuous	1.2	1.1–1.3	**<0.001**
AST level	Continuous	1.1	1.0–1.2	**0.005**
INR level	Continuous	1.0	0.9–1.1	0.686
Tumor number	Continuous	1.1	1.0–1.2	**0.044**
Max. lesion size	Continuous	1.3	1.1–1.5	**<0.001**
SM/Bone	Continuous	0.9	0.8–1.0	**0.036**
TAT/Bone	Continuous	1.0	0.9–1.1	0.985
**Sarcopenia marker**				
AFP	Continuous	1.0	1.0–1.1	0.309
Albumin	Continuous	0.6	0.5–0.7	**<0.001**
Bilirubin	Continuous	1.2	1.1–1.3	**<0.001**
AST level	Continuous	1.1	1.0–1.2	**0.006**
INR level	Continuous	1.0	0.9–1.1	0.694
Tumor number	Continuous	1.1	1.0–1.2	**0.044**
Max. lesion size	Continuous	1.3	1.1–1.5	**<0.001**
Sarcopenia marker	Continuous	0.9	0.8–1.0	**0.030**

p values in bold show significant values.
AFP, alpha fetoprotein; ALT, alanine aminotransferase; AST, aspartate aminotransferase; IMAT, intermuscular adipose tissue; INR, international normalized ratio; SAT, subcutaneous adipose tissue; SM, skeletal muscle volume; TAT, total adipose tissue; VAT, visceral adipose tissue.
*Volumes normalized to the slice number of the abdominal cavity.

relevant prognostic factor in their cohort. These results seem remarkable for several reasons. First, they contradict the hypothesis that the reserves a patient has at the start of therapy are relevant to prognosis. Second, they indicate that TACE, as a locoregional therapy, seems to have a systematic impact on BCA parameters, despite its selective application. However, the study by Fujita *et al.* included only a moderate number of patients (n = 179) and data from a single center. Another study by Zheng *et al.* included 75 patients, and exclusively examined the changes of BCA parameters during treatment. They quantified the body composition parameters using a semi-automatic method, with necessary manual input, and found that changes in SM, SAT, and VAT all remained independent prognostic factors in multivariate Cox regression analysis.[18]

When comparing the results of our present study with previous works, it is important to note that, to our knowledge, all previous studies investigating patients with HCC and TACE have exclusively focused on patients from Asian cohorts. In addition to natural differences in body constitution due to ethnicity, it is possible that the results are influenced by another prognostic role due to the origin of cirrhosis. Notably, in Europe, alcohol-related liver disease with cirrhotic remodeling of the tissue accounts for a larger proportion of patients in most HCC patient cohorts. On the other hand, in Asian cohorts, HCC in the non-cirrhotic liver is commonly the result of chronic hepatitis B. This etiological difference could potentially have a considerable influence on the prognostic role of BCA parameters in patients with HCC. Patients with HCC and cirrhosis have two diseases in parallel, and cirrhosis, as a preexisting chronic disease, has a strong influence on body constitution.[40]

Compared to previous studies in this field, apart from its multicentric character and the fully automated AI-based BCA, another strength of our study is that the patients had not received any previous treatment. This design was chosen to increase homogeneity. Nevertheless, follow-up studies could investigate patients with previous therapies. For deciding whether TACE should be initiated in certain cases, it could be of particular interest how the BCA parameters are influenced by previous treatment courses. However, these are often individualized courses of therapy, which would require a higher number of included patients for such subgroup analysis to be representative.

To the best of our knowledge, there are no studies on automated 3D-based BCA in patients with HCC. Due to the

Table 5. Univariate and multivariate Cox regression analysis for all patients (categorized variables) (N = 754).

Covariate		Univariate analysis HR	95% CI	*p* value	Multivariate analysis HR	95% CI	*p* value
Age	≥70 years	0.9	0.8–1.1	0.230			
AFP	>400 ng/ml	1.0	0.9–1.3	0.720			
Albumin	<35 g/L	2.1	1.7–2.5	**<0.001**	1.8	1.5–2.3	**<0.001**
Bilirubin	≥1.2 mg/dl	1.8	1.5–2.2	**<0.001**	1.6	1.3–2.0	**<0.001**
AST level	>31 U/L	1.4	1.0–1.9	0.054			
ALT level	≥35 U/L	1.1	0.9–1.3	0.430			
INR level	>1.2	0.7	0.6–0.9	**<0.001**	1.1	0.8–1.3	0.665
Platelet count	<150/nl	1.1	0.9–1.3	0.500			
Tumor number	≥2	1.4	1.1–1.7	**0.001**	1.2	1.0–1.5	**0.048**
Max. lesion size	>5.0 cm	1.6	1.3–2.0	**<0.001**	1.6	1.3–2.0	**<0.001**
SM normalized*	<89.0	1.6	1.2–2.0	**<0.001**	1.4	1.1–1.8	**0.019**
TAT normalized*	<197.6	0.6	0.5–0.8	**<0.001**	0.8	0.6–1.2	0.269
IMAT normalized*	<18.8	0.8	0.7–1.0	0.052			
SAT normalized*	<119.5	0.7	0.5–0.8	**<0.001**	1.0	0.7–1.3	0.774
VAT normalized*	<58.2	0.8	0.7–1.0	**0.030**	1.1	0.9–1.4	0.497

p values in bold show significant values.
AFP, alpha fetoprotein; ALT, alanine aminotransferase; AST, aspartate aminotransferase; HR, hazard ratio; IMAT, intermuscular adipose tissue; INR, international normalized ratio; SAT, subcutaneous adipose tissue; SM, skeletal muscle volume; TAT, total adipose tissue; VAT, visceral adipose tissue.
*Volumes normalized to the slice number of the abdominal cavity.

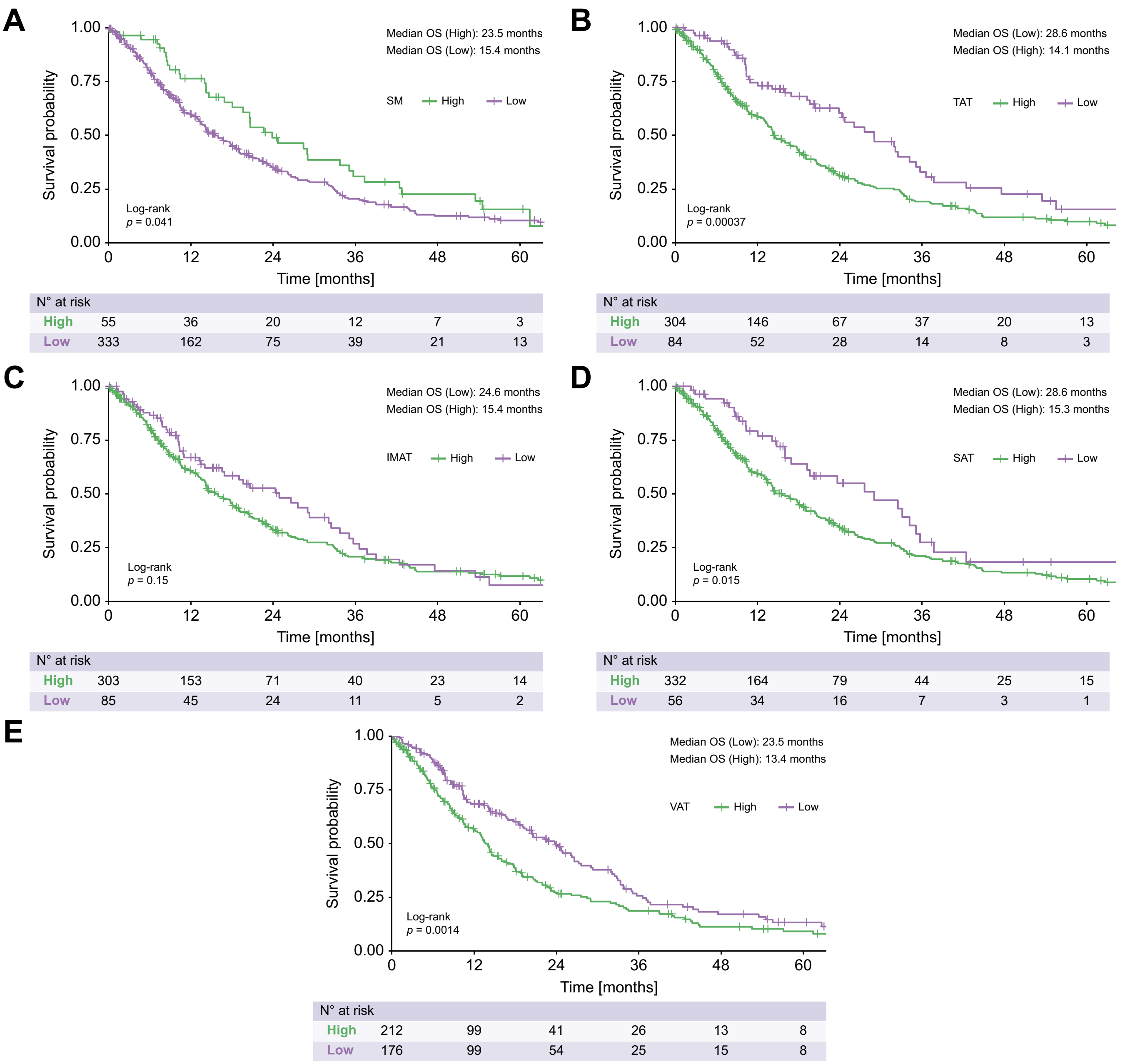

Fig. 3. Kaplan-Meier curves for the overall survival of the BCLC stage B patients, stratified according to their BCA parameters. All log rank test, *p* values: A 0.041, B <0.001, C = 0.150, D = 0.015, E = 0.001). BCA, body composition assessment; BCLC, Barcelona Clinic Liver Cancer; IMAT, intermuscular adipose tissue; SAT, subcutaneous adipose tissue; SM, skeletal muscle volume; TAT, total adipose tissue; VAT, visceral adipose tissue.

fundamental methodological differences and the above-mentioned weaknesses of a 2D measurement, comparability with existing data is only possible indirectly. Nevertheless, in order to improve comparability of the results, we additionally evaluated a biomarker proposed for other patient cohorts with hepatobiliary malignancies or liver diseases and were able to confirm the predictive role of the MBR (muscle-to-bone ratio) and the proposed sarcopenia marker for our patient cohort.[31–33] In a subgroup of patients with available body height, we were also able to confirm the relation of SM/body height as an independent prognostic factor in our study.

Notably, our data show a trend towards overlap of BCA risk groups in the long term. In our study, however, we only examined the baseline BCA parameters. These only reflect long-term changes to a limited extent. In particular, the influence of oncological therapy on BCA parameters and changes in BCA parameters may have prognostic relevance, as BCA parameters are so-called time-dependent risk factors.[41] Future studies with examination at different time points are necessary to investigate the influence of BCA parameters in the long-term course of therapy.

It is worth mentioning that poor muscle status of a patient alone should not be used to guide treatment decisions. However, quantification of this opportunistic imaging parameter could in the future ensure that currently used semi-quantitative parameters such as ECOG status are supported by quantitative

Table 6. Univariate and multivariate Cox regression analysis for patients within BCLC stage B (n = 388).

Covariate		Univariate analysis			Multivariate analysis		
		HR	95% CI	*p* value	HR	95% CI	*p* value
Age	≥70 years	1.0	0.8–1.2	0.720			
AFP	>400 ng/ml	1.1	0.9–1.5	0.370			
Albumin	<35 g/L	1.9	1.5–2.4	**<0.001**	1.7	1.3–2.2	**<0.001**
Bilirubin	≥1.2 mg/dl	1.8	1.4–2.2	**<0.001**	1.5	1.1–2.0	**0.005**
AST level	>31 U/L	1.3	0.9–2.1	0.220			
ALT level	≥35 U/L	1.0	0.8–1.4	0.750			
INR level	>1.2	0.6	0.5–0.8	**<0.001**	1.0	0.7–1.3	0.748
Platelet count	<150/nl	1.2	0.9–1.6	0.210			
Tumor number	≥2	1.0	0.7–1.5	0.870			
Max. lesion size	>5.0 cm	1.2	1.0–1.6	0.110			
SM normalized*	<89.0	1.4	1.0–2.1	**0.041**	1.6	1.1–2.3	**0.012**
TAT normalized*	<197.6	0.6	0.4–0.8	**<0.001**	0.8	0.5–1.2	0.287
IMAT normalized*	<18.8	0.8	0.6–1.1	0.150			
SAT normalized*	<119.5	0.6	0.4–0.9	**0.016**	0.8	0.5–1.1	0.523
VAT normalized*	<58.2	0.7	0.5–0.9	**0.001**	0.9	0.6–1.1	0.289

p values in bold show significant values.
AFP, alpha fetoprotein; ALT, alanine aminotransferase; AST, aspartate aminotransferase; HR, hazard ratio; IMAT, intermuscular adipose tissue; INR, international normalized ratio; SAT, subcutaneous adipose tissue; SM, skeletal muscle volume; TAT, total adipose tissue; VAT, visceral adipose tissue.
*Volumes normalized to the slice number of the abdominal cavity.

and reproducible data. Especially as imaging data is available for every patient due to treatment planning issues and fully automated approaches make BCA parameters available as opportunistic imaging biomarkers without any additional effort. Thus, clinical decision-making processes could be enhanced with additional quantitative data.

In this study, OS was selected as the primary endpoint, with patient recruitment spanning from 2010 to 2020. During this timeframe, treatment options post-TACE ineligibility mainly relied on sorafenib and lenvatinib use. However, only a minority of patients received systemic treatment after TACE failure.[42] The emergence of novel immunotherapeutic options post-TACE ineligibility challenges the suitability of OS assessment in TACE studies, as more patients could qualify for an early treatment switch towards highly effective immunotherapeutic agents, potentially impacting future post-TACE survival rates. Despite this, there is insufficient evidence backing alternative endpoints in liver cancer trials, especially in patients undergoing TACE. Progression-free survival and time to progression may inadequately capture the clinical reality of these patients.[43] While composite endpoints (*e.g.* "failure of strategy" (NCT04803994)) are employed in clinical trials, they are not viable for retrospective analysis. Future research is warranted to explore alternative endpoints for liver trials and their correlation with survival as the only endpoint with "absolute precision".[43]

Our present study had several limitations. First, the analysis was performed using a retrospectively collected dataset. It would be beneficial to validate the current findings in a prospective setting. Second, our study cohort comprised patients in BCLC stages 0, A, B, and C. While TACE is the recommended standard treatment for patients with intermediate-stage disease (BCLC stage B), our cohort reflects the real-world clinical practice in most countries. TACE is commonly administered in more advanced or earlier stages, following the concept of stage migration, which has been endorsed by the EASL guidelines.[2] Notably, we also conducted a subgroup analysis specifically among the patients with BCLC stage B, for whom TACE is the recommended standard treatment. Third, the analysis of BCA was performed using imaging datasets from different institutions, such that the scans had been acquired on different scanner types. Despite visual inspection and normalization steps – such as uniform scaling of slice thickness – the influence of different scanner types is difficult to estimate in our current study design. However, preliminary work from OSCAR (Opportunistic Screening Consortium in Abdominal Radiology) indicates that mature algorithms, such as the one we used, can be expected to make very few errors in the analysis of even heterogeneous external datasets.[44] Fourth, we did not perform any subgroup analysis of patients treated using different TACE techniques. However, multiple comparisons between conventional TACE and drug-eluting bead TACE have not revealed any influence on OS.[45–47] In this study, we have tried to include as many factors as possible that influence the TACE procedure. However, there are a number of other confounding factors that would need to be controlled for in a prospective setting.

Our present results revealed that skeletal muscle volume was an independent prognostic factor for survival prediction in patients with HCC undergoing TACE. Thus, the integration of this parameter into novel scoring systems could potentially improve survival prediction and clinical decision-making. Fully automated approaches are essential for fostering the implementation of this imaging biomarker into daily routine practice. Incorporating AI-based solutions through a fully automated approach holds the potential to seamlessly integrate BCA into the regular clinical workflow for patients with HCC undergoing TACE. This strategy could effectively overcome the methodological challenges that currently limit the widespread adoption of BCA for opportunistic CT screening in all patients.

Affiliations
[1]*Department of Diagnostic and Interventional Radiology, University Medical Center of the Johannes Gutenberg University Mainz, Mainz, Germany;* [2]*Department of Radiology, Charité - University Medicine Berlin, Berlin, Germany;* [3]*Berlin Institute of Health at Charité - University Medicine Berlin, Berlin, Germany;* [4]*Department of Diagnostic and Interventional Radiology and Neuroradiology, University Hospital Essen, Essen, Germany;* [5]*Institute for AI in Medicine (IKIM), University Hospital Essen, Essen, Germany;* [6]*Department of Diagnostic and Interventional Radiology, Freiburg University Hospital, Freiburg, Germany;* [7]*Department of Diagnostic and*

Interventional Radiology, Medical Faculty OWL, Bielefeld University, Bielefeld, Germany; [8]Department of Medicine II, Freiburg University Hospital, Freiburg, Germany; [9]Department of Diagnostic and Interventional Radiology, University Medical Center Heidelberg, Heidelberg, Germany; [10]Department of Radiology, University Hospital Cologne, Cologne, Germany; [11]Department of Radiology, University Hospital of Frankfurt, Frankfurt, Germany; [12]Institute of Interventional Radiology, University Hospital of Schleswig-Holstein – Campus Lübeck, Lübeck, Germany

Abbreviations

AI, artificial intelligence; BCA, body composition assessment; BCLC, Barcelona Clinic Liver Cancer; CT, Computed Tomography; EASL, the European Association for the Study of the Liver; HCC, hepatocellular carcinoma; HR, hazard ratio; IMAT, intermuscular adipose tissue; OS, overall survival; SAT, subcutaneous adipose tissue; SM, skeletal muscle volume; TACE, transarterial chemoembolization; TAT, total abdominal adipose tissue; VAT, visceral adipose tissue.

Financial support

This study was not supported by any sponsor or funder.

Conflict of interest

The authors have no conflicts of interest to declare.

Please refer to the accompanying ICMJE disclosure forms for further details.

Authors' contributions

L.M., A.M.K., T.A.A., U.F., B.G., J.H., B.M.S., M.-S.K., R.H., F.N., J.K., T.D.D., M.E., H.K., N.R., D.B., V.S., P.M., D.Z., D.P.D.S., and R.K. devised the study, assisted in data collection, participated in the interpretation of the data, and helped draft the manuscript. L.M., T.A.A., U.F., J.H., M.-S.K, T.D.D., N.R., D.B., V.S., D.Z., and R.K. carried out the data collection. D.P.D.S., P.M., H.K., M.E., R.H., F.N., J.K., B.M.S., B.G., and A.M.K. supported the data collection efforts. L.M., A.M.K., D.P.D.S., and R.K. created all of the figures and participated in the interpretation of data. L.M., A.M.K., D.P.D.S., and R.K. performed the statistical analysis. All authors contributed to the article and approved the submitted version.

Data availability statement

The datasets presented in this article are not readily available because data cannot be shared publicly because of institutional and national data policy restrictions imposed by the local Ethics. Data are available upon request for researchers who meet the criteria for access to confidential data. Requests to access the datasets should be directed to roman.kloeckner@uksh.de or lukas.mueller@unimedizin-mainz.de.

Acknowledgements

L.M. is supported by the Clinician Scientist Fellowship "Else Kröner Research College: 2018_Kolleg.05".

Supplementary data

Supplementary data to this article can be found online at https://doi.org/10.1016/j.jhepr.2024.101125.

References

[1] Bray F, Ferlay J, Soerjomataram I, et al. Global cancer statistics 2018: GLOBOCAN estimates of incidence and mortality worldwide for 36 cancers in 185 countries. CA Cancer J Clin 2018;68:394–424.
[2] Galle PR, Forner A, Llovet JM, et al. EASL clinical practice guidelines: management of hepatocellular carcinoma. J Hepatol 2018;69:182–236. https://doi.org/10.1016/j.jhep.2018.03.019.
[3] Heimbach JK, Kulik LM, Finn RS, et al. AASLD guidelines for the treatment of hepatocellular carcinoma. Hepatology 2018;67:358–380. https://doi.org/10.1002/hep.29086.
[4] Raoul J-L, Forner A, Bolondi L, et al. Updated use of TACE for hepatocellular carcinoma treatment: how and when to use it based on clinical evidence. Cancer Treat Rev 2019;72:28–36. https://doi.org/10.1016/j.ctrv.2018.11.002.
[5] Bolondi L, Burroughs A, Dufour J-F, et al. Heterogeneity of patients with intermediate (BCLC B) Hepatocellular Carcinoma: proposal for a subclassification to facilitate treatment decisions. In: Seminars in liver disease. Thieme Medical Publishers; 2012. p. 348–359.
[6] Park J, Chen M, Colombo M, et al. Global patterns of hepatocellular carcinoma management from diagnosis to death: the BRIDGE Study. Liver Int 2015;35:2155–2166.
[7] Müller L, Stoehr F, Mähringer-Kunz A, et al. Current strategies to identify patients that will benefit from TACE treatment and future directions a practical step-by-step guide. J Hepatocell Carcinoma 2021;8:403.
[8] Mähringer-Kunz A, Wagner F, Hahn F, et al. Predicting survival after transarterial chemoembolization for hepatocellular carcinoma using a neural network: a Pilot Study. Liver Int 2020;40:694–703. https://doi.org/10.1111/liv.14380.
[9] Chang K-V, Chen J-D, Wu W-T, et al. Association between loss of skeletal muscle mass and mortality and tumor recurrence in hepatocellular carcinoma: a systematic review and meta-analysis. Liver cancer 2018;7:90–103.
[10] Hahn F, Müller L, Stöhr F, et al. The role of sarcopenia in patients with intrahepatic cholangiocarcinoma: prognostic marker or hyped parameter? Liver Int 2019;39:1307–1314. https://doi.org/10.1111/liv.14132.
[11] Jiang C, Wang Y, Fu W, et al. Association between sarcopenia and prognosis of hepatocellular carcinoma: a systematic review and meta-analysis. Front Nutr 2022;9:978110.
[12] Fujita M, Takahashi A, Hayashi M, et al. Skeletal muscle volume loss during transarterial chemoembolization predicts poor prognosis in patients with hepatocellular carcinoma. Hepatol Res 2019;49:778–786.
[13] Chien T-P, Huang S-F, Chan W-H, et al. The combination of sarcopenia and biochemical factors can predict the survival of hepatocellular carcinoma patients receiving transarterial chemoembolization. Front Oncol 2022;12:1005571.
[14] Loosen SH, Jördens MS, Schoon B, et al. Sarcopenia indicate poor survival in patients undergoing transarterial chemoembolization (TACE) for hepatic malignancies. J Cancer Res Clin Oncol 2023:1–10.
[15] Bannangkoon K, Hongsakul K, Tubtawee T, et al. Association of myosteatosis with treatment response and survival in patients with hepatocellular carcinoma undergoing chemoembolization: a retrospective cohort study. Sci Rep 2023;13:3978.
[16] Li Y, Hou J, Chen R. Prognostic value of sarcopenic visceral obesity in hepatocellular carcinoma treated with TACE. Medicine (Baltimore) 2023;102.
[17] Zhang J-X, Yan H-T, Ding Y, et al. Low Psoas-Muscle index is associated with decreased survival in hepatocellular carcinoma treated with transarterial chemoembolization. Ann Med 2022;54:1562–1569.
[18] Zheng X, Cao F, Qian L, et al. Body composition changes in hepatocellular carcinoma: prediction of survival to transcatheter arterial chemoembolization in combination with clinical prognostic factors. Cancer Control 2021;28:10732748211038444.
[19] Pickhardt PJ. Value-added opportunistic CT screening: state of the art. Radiology 2022;303:241–254.
[20] Li Q, Zhang L, Hou Z-H, et al. High visceral adipose tissue density correlates with unfavorable outcomes in patients with intermediate-stage hepatocellular carcinoma undergoing transarterial chemoembolization. Front Cel Dev Biol 2021;9:710104.
[21] Koitka S, Kroll L, Malamutmann E, et al. Fully automated body composition analysis in routine CT imaging using 3D semantic segmentation convolutional neural networks. Eur Radiol 2020. https://doi.org/10.1007/s00330-020-07147-3.
[22] Collins GS, Reitsma JB, Altman DG, et al. Transparent reporting of a multivariable prediction model for individual prognosis or diagnosis (TRIPOD) the TRIPOD statement. Circulation 2015;131:211–219.
[23] Müller L, Hahn F, Mähringer-Kunz A, et al. Immunonutritive scoring in patients with hepatocellular carcinoma undergoing transarterial chemoembolization: prognostic nutritional index or controlling nutritional status score? Front Oncol 2021;11:2205.
[24] Auer TA, Müller L, Schulze D, et al. CT-guided high-dose-rate brachytherapy versus transarterial chemoembolization in patients with unresectable hepatocellular carcinoma. Radiology 2024;310:e232044.
[25] Lammer J, Malagari K, Vogl T, et al. Prospective randomized study of doxorubicin-eluting-bead embolization in the treatment of hepatocellular carcinoma: results of the PRECISION V study. Cardiovasc Intervent Radiol 2010;33:41–52. https://doi.org/10.1007/s00270-009-9711-7.
[26] Lencioni R, De Baere T, Burrel M, et al. Transcatheter treatment of hepatocellular carcinoma with Doxorubicin-loaded DC Bead (DEBDOX): technical recommendations. Cardiovasc Intervent Radiol 2012;35:980–985.
[27] Auer TA, Jonczyk M, Collettini F, et al. Trans-arterial chemoembolization with degradable starch microspheres (DSM-TACE) versus selective internal radiation therapy (SIRT) in multifocal hepatocellular carcinoma. Acta Radiol 2021;62:313–321.
[28] Lencioni R, Llovet JM. Modified RECIST (mRECIST) assessment for hepatocellular carcinoma. In: Seminars in liver disease. © Thieme Medical Publishers; 2010. p. 52–60.

[29] Scherer J, Nolden M, Kleesiek J, et al. Joint imaging Platform for federated clinical data analytics. JCO Clin Cancer Inform 2020:1027–1038. https://doi.org/10.1200/CCI.20.00045.
[30] Hosch R, Kattner S, Berger MM, et al. Biomarkers extracted by fully automated body composition analysis from chest CT correlate with SARS-CoV-2 outcome severity. Sci Rep 2022;12:16411.
[31] Keyl J, Hosch R, Berger A, et al. Deep learning-based assessment of body composition and liver tumour burden for survival modelling in advanced colorectal cancer. J Cachexia Sarcopenia Muscle 2023;14:545–552.
[32] Keyl J, Bucher A, Jungmann F, et al. Prognostic value of deep learning-derived body composition in advanced pancreatic cancer—a retrospective multicenter study. ESMO open 2024;9:102219.
[33] Alatzides GL, Haubold J, Steinberg HL, et al. Adipopenia in body composition analysis: a promising imaging biomarker and potential predictive factor for patients undergoing transjugular intrahepatic portosystemic shunt placement. Br J Radiol 2023;96:20220863.
[34] Kappal S. Data normalization using median absolute deviation MMAD based Z-score for robust predictions *vs.* min–max normalization. Lond J Res Sci Nat Form 2019;19:10–13140.
[35] Borde T, Nezami N, Gaupp FL, et al. Optimization of the BCLC staging system for locoregional therapy for hepatocellular carcinoma by using quantitative tumor burden imaging biomarkers at MRI. Radiology 2022:212426.
[36] Stoehr F, Kloeckner R, Pinto dos Santos D, et al. Radiomics-based prediction of future portal vein tumor infiltration in patients with HCC—a proof-of-concept study. Cancers (Basel) 2022;14:6036.
[37] Fujiwara N, Nakagawa H, Kudo Y, et al. Sarcopenia, intramuscular fat deposition, and visceral adiposity independently predict the outcomes of hepatocellular carcinoma. J Hepatol 2015;63:131–140.
[38] Müller L, Mähringer-Kunz A, Auer TA, et al. Low bone mineral density is a prognostic factor for elderly patients with HCC undergoing TACE: results from a multicenter study. Eur Radiol 2022. https://doi.org/10.1007/s00330-022-09069-8.
[39] Royston P, Altman DG, Sauerbrei W. Dichotomizing continuous predictors in multiple regression: a bad idea. Stat Med 2006;25:127–141.
[40] Lai JC, Tandon P, Bernal W, et al. Malnutrition, frailty, and sarcopenia in patients with cirrhosis: 2021 practice guidance by the American Association for the Study of Liver Diseases. Hepatology 2021;74:1611.
[41] Dekker FW, De Mutsert R, Van Dijk PC, et al. Survival analysis: time-dependent effects and time-varying risk factors. Kidney Int 2008;74:994–997.
[42] Peck-Radosavljevic M, Kudo M, Raoul J-L, et al. Outcomes of patients (pts) with hepatocellular carcinoma (HCC) treated with transarterial chemoembolization (TACE): global OPTIMIS final analysis. J Clin Oncol 2018;36:4018. https://doi.org/10.1200/JCO.2018.36.15_suppl.4018.
[43] Bruix J. Endpoints in clinical trials for liver cancer and their value in evidence-based clinical decision making: an unresolved Gordian knot. J Hepatol 2021;74:1483–1488.
[44] Pooler BD, Garrett JW, Southard AM, et al. Technical adequacy of fully automated artificial intelligence body composition tools: assessment in a heterogeneous sample of external CT examinations. Am J Roentgenol 2023:1–9.
[45] Sacco R, Bargellini I, Bertini M, et al. Conventional versus doxorubicin-eluting bead transarterial chemoembolization for hepatocellular carcinoma. J Vasc Interv Radiol 2011;22:1545–1552. https://doi.org/10.1016/j.jvir.2011.07.002.
[46] Golfieri R, Giampalma E, Renzulli M, et al. Randomised controlled trial of doxorubicin-eluting beads vs conventional chemoembolisation for hepatocellular carcinoma. Br J Cancer 2014;111:255–264. https://doi.org/10.1038/bjc.2014.199.
[47] Kloeckner R, Weinmann A, Prinz F, et al. Conventional transarterial chemoembolization versus drug-eluting bead transarterial chemoembolization for the treatment of hepatocellular carcinoma. BMC Cancer 2015;15:465. https://doi.org/10.1186/s12885-015-1480-x.

Keywords: Hepatocellular Carcinoma; Artificial Intelligence; Transarterial Chemoembolization; Body Composition.
Received 21 October 2023; received in revised form 17 May 2024; accepted 23 May 2024; Available online 25 May 2024

Journal of Hepatology, Volume *6*

Supplemental information

AI-derived body composition parameters as prognostic factors in patients with HCC undergoing TACE in a multicenter study

Lukas Müller, Aline Mähringer-Kunz, Timo Alexander Auer, Uli Fehrenbach, Bernhard Gebauer, Johannes Haubold, Benedikt Michael Schaarschmidt, Moon-Sung Kim, René Hosch, Felix Nensa, Jens Kleesiek, Thierno D. Diallo, Michel Eisenblätter, Hanna Kuzior, Natascha Roehlen, Dominik Bettinger, Verena Steinle, Philipp Mayer, David Zopfs, Daniel Pinto Dos Santos, and Roman Kloeckner

AI-derived body composition parameters as prognostic factors in patients with HCC undergoing TACE in a multicenter study

Lukas Müller, Aline Mähringer-Kunz, Timo Alexander Auer, Uli Fehrenbach, Bernhard Gebauer, Johannes Haubold, Benedikt Michael Schaarschmidt, Moon-Sung Kim, René Hösch, Felix Nensa, Jens Kleesiek, Thierno D. Diallo, Michel Eisenblätter, Hanna Kuzior, Natascha Roehlen, Dominik Bettinger, Verena Steinle, Philipp Mayer, David Zopfs, Daniel Pinto Dos Santos, Roman Kloeckner

Table of contents

Table S1……………………………………………………………………………………2

Table S2……………………………………………………………………………………4

Table S3……………………………………………………………………………………6

Table S4……………………………………………………………………………………8

Table S5…………………………………………………………………………………..10

Table S6…………………………………………………………………………………..11

Table S7…………………………………………………………………………………..12

Table S8…………………………………………………………………………………..13

Table S9…………………………………………………………………………………..14

Table S10…………………………………………………………………………………15

Fig. S1……………………………………………………………………………………18

Fig. S2……………………………………………………………………………………19

Table S1. Baseline characteristics stratified by sex

**Variable**	**Male (*n* = 622)**	**Female (n=132)**	**p value**
Age in years, median (IQR)	67 (60–74)	67 (61 – 74)	0.682
Etiology of liver cirrhosis, *n* (%)			<0.001
Alcohol	283	29	
Viral	171	48	
Other	97	44	
Child-Pugh stage, *n* (%)			0.812
No cirrhosis	70	11	
A	322	71	
B	229	50	
BCLC stage, *n* (%)			0.263
0	8	2	
A	177	42	
B	316	72	
C	121	16	
Size of the largest lesion in mm, median (IQR)	38 (26 – 62)	39 (27 – 59)	0.967
Number of lesions, median (IQR)	1 (1 – 4)	2 (1 – 3)	0.627
Albumin level, g/L, median (IQR)	35 (30 – 39)	36 (30 – 39)	0.634

Bilirubin level, mg/dL, median (IQR)	1.1 (0.7 – 1.8)	1.3 (0.8 – 2.0)	0.105
Platelet count, per nL, median (IQR)	126 (85 – 190)	111 (77 – 180)	0.177
AST level, U/L, median (IQR)	59.0 (42.0 – 90.0)	68.5 (49.5 – 106.0)	0.007
ALT level, U/L, median (IQR)	40.0 (27.0 – 62.0)	41.0 (26.0 – 65.5)	0.662
INR, median (IQR)	1.2 (1.1 – 1.3)	1.2 (1.0 – 1.3)	0.494
AFP level, ng/mL, median (IQR)	21.7 (5.7 – 277.5)	44.0 (7.9 – 1015.0)	0.017

Comparison using Fisher test or Chi^2 for categorial variables and Mann-Whitney for continuous variables. P-values in bold show significant values.

Table S2. Baseline characteristics stratified by BCLC stage

**Variable**	**BCLC stage A (n=219)**	**BCLC stage B (n=388)**	**BCLC stage C (n=137)**	**p value**
Age in years, median (IQR)	65 (60 – 72)	68 (61 – 74)	66 (58 – 75)	0.033
Sex, *n* (%)				0.263
Female	42 (19.2)	72 (18.6)	16 (12.4)	
Male	177 (80.8)	316 (81.4)	121 (87.6)	
Etiology of liver cirrhosis, *n* (%)				0.047
Alcohol	100	162	48	
Viral	73	101	41	
Other	29	77	31	
Child-Pugh stage, *n* (%)				<0.001
No cirrhosis	17	48	17	
A	133	195	59	
B	69	145	61	
Size of the largest lesion in mm, median (IQR)	29 (23 – 39)	47 (33 – 65)	56 (33 – 87)	<0.001
Number of lesions, median (IQR)	1 (1 – 2)	3 (2 – 4)	2 (1 – 3)	<0.001
Albumin level, g/L, median (IQR)	36 (31 – 40)	35 (13 – 39)	32 (27 – 37)	<0.001
Bilirubin level, mg/dL, median (IQR)	1.1 (0.7 – 1.8)	1.2 (0.7 – 1.8)	1.2 (0.7 – 2.1)	0.812

Platelet count, per nL, median (IQR)	106 (77 – 165)	120 (84 – 194)	154 (105 – 234)	<0.001
AST level, U/L, median (IQR)	52.0 (38.0 – 70.0)	63.0 (44.3 – 94.8)	79.5 (51.3 – 114)	<0.001
ALT level, U/L, median (IQR)	38.0 (24.3 – 57.0)	41.0 (28.0 – 63.0)	41.0 (28.0 – 66.8)	0.266
INR, median (IQR)	1.2 (1.1 – 1.3)	1.1 (1.1 – 1.3)	1.2 (1.1 – 1.3)	0.242
AFP level, ng/mL, median (IQR)	10.4 (4.4 – 50.8)	33.6 (6.4 – 349.7)	148.0 (10.3 – 2639.7)	<0.001

Comparison using Fisher test or Chi^2 for categorial variables and Mann-Whitney for continuous variables. P-values in bold show significant values.

Table S3. BCA parameters stratified by sex

**Variable**	**Male (*n* = 622)**	**Female (n=132)**	**p value**
SM, mL	6160.1 (3468.6 – 7526.8)	4826.9 (2746.5 – 5900.4)	<0.001
TAT, mL	10904.8 (7636.3 – 15337.7	9640.0 (6965.0 – 15277.8)	0.231
IMAT, mL	922.3 (593.2 – 1493.5)	890.2 (523.3 – 1397.0)	0.284
SAT, mL	5495.2 (3573.3 – 8003.9)	6300.8 (4315.5 – 9942.4)	0.002
VAT, mL	4195.5 (2846.8 – 5878.4)	2400.1 (1667.4 – 3701.4)	<0.001
SM normalized*	74.1 (62.7 – 85.7)	59.6 (51.6 – 68.1)	<0.001
TAT normalized*	154.9 (112.3 – 197.3)	145.8 (106.8 – 189.3)	0.154
IMAT normalized*	12.8 (9.2 – 17.7)	11.3 (8.4 – 15.8)	0.121
SAT normalized*	74.2 (54.2 – 99.3)	92.3 (63.5 – 120.9)	<0.001
VAT normalized*	59.0 (39.3 – 80.6)	36.5 (23.4 – 50.7)	<0.001
SM/Bone	2.5 (2.2 – 2.8)	2.4 (2.1 – 2.7)	0.011
TAT/Bone	5.3 (3.8 – 6.9)	6.0 (4.2 – 7.6)	0.013
IMAT/Bone	0.4 (0.3 – 0.6)	0.5 (0.4 – 0.6)	0.068
SAT/Bone	2.6 (1.9 – 3.4)	3.8 (2.5 – 5.0)	<0.001

VAT/Bone	2.0 (1.3 – 2.8)	1.4 (1.0 – 2.1)	<0.001
Sarcopenia marker	1.7 (1.5 – 2.0)	1.6 (1.4 – 1.9)	0.002

Comparison using Mann-Whitney test. P-values in bold show significant values.

Table S4. BCA parameters stratified by BCLC stages

Variable	BCLC stage A	BCLC stage B	BCLC stage C	Overall p value
SM, mL	6018.9 (3978.4 – 7595.7)	5604.3 (3160.9 – 7359.5)	5510.7 (3352.3 – 7 241.2)	0.139
TAT, mL	11089.6 (7768.3 – 15844.3)	10986.1 (7618.2 – 15083.0)	10179.6 (6885.5 – 15068.0)	0.605
IMAT, mL	893.9 (620.5 – 1452.0)	944.6 (579.5 – 1456.4)	874.3 (557.6 – 1442.7)	0.819
SAT, mL	5942.8 (3988.1 – 8425.3)	5575.9 (3703.1 – 8265.3)	4981.3 (3496.5 – 7407.2)	0.219
VAT, mL	3685.0 (2409.7 – 5814.8)	3972.2 (2652.1 – 5404.4)	3913.6 (2444.6 – 5560.2)	0.950
SM normalized*	74.1 (61.1 – 87.5)	70.6 (59.0 – 82.5)	69.9 (60.6 – 81.8)	0.135
TAT normalized*	151.5 (106.7 – 204.3)	155.3 (113.8 – 190.7)	150.2 (114.4 – 190.0)	0.894
IMAT normalized*	12.1 (8.7 – 16.8)	13.0 (9.2 – 17.7)	12.4 (9.1 – 16.8)	0.710

SAT normalized*	76.7 (56.2 – 110.6)	78.1 (55.9 – 103.0)	72.7 (54.3 – 94.7)	0.460
VAT normalized*	55.4 (33.2 – 74.3)	54.1 (37.4 – 77.8)	56.2 (37.2 – 76.8)	0.722
SM/Bone	2.6 (2.2 – 2.9)	2.5 (2.2 – 2.8)	2.5 (2.2 – 2.8)	0.196
TAT/Bone	5.3 (3.7 – 7.2)	5.4 (4.0 – 7.1)	5.3 (3.8 – 6.7)	0.853
IMAT/Bone	0.43 (0.32 – 0.58)	0.45 (0.34 – 0.62)	0.45 (0.32 – 0.58)	0.462
SAT/Bone	2.7 (1.9 – 3.9)	2.7 (2.0 – 3.8)	2.5 (2.0 – 3.5)	0.568
VAT/Bone	1.8 (1.1 – 2.7)	1.9 (1.3 – 2.7)	2.0 (1.4 – 2.6)	0.570
Sarcopenia marker	1.8 (1.5 – 2.1)	1.7 (1.5 – 2.0)	1.7 (1.4 – 2.0)	0.187

All data are presented as median (IQR).

SM, skeletal muscle volume; TAT, total adipose tissue; IMAT, intermuscular adipose tissue; VAT, visceral adipose tissue; SAT, subcutaneous adipose tissue. *Volumes normalized to the slice number of the abdominal cavity. Kruskal-Wallis test for comparison. P-values in bold show significant values.

Table S5. Technical details of the performed TACE procedures

**Variable**	**All patients (*n* = 754)**
Type of TACE, *n* (%)	
cTACE	392 (52.0)
DEB-TACE	348 (46.2)
DSM-TACE	14 (1.8)
Level of selectivity, *n* (%)	
Selective	167 (22.1)
Superselective	587 (77.9)
Use of CBCT, *n* (%)	
Yes	69 (9.2)
No	685 (90.8)
Type of drug*, n	
Doxirubicin	390
Mitomycin	315
Epirubicin	277
Cisplatin	8
Irinotecan	1

cTACE, conventional transarterial chemoembolization; DEB-TACE, drug-eluting beads transarterial chemoembolization; DSM-TACE, degradable starch microspheres transarterial chemoembolization; CBCT, cone-beam computed tomography. *More than one drug possible.

Table S6. Correlation SM with other significant factors in multivariate analysis

**Variables**	**Correlation coefficient**	**Interpretation correlation**	**p value**
Albumin	0.168	Weak	**<0.001**
Bilirubin	-0.068	Negligible	0.062
AST	-0.030	Negligible	0.436
Max. lesion size	-0.075	Negligible	**0.047**

Interpretation: 10.1213/ANE.0000000000002864. Spearman correlation. P-values in bold show significant values.

Table S7. Univariate Cox regression analysis for additional clinical, laboratory and tumor burden-related variables apart from BCA parameters in all patients (continuous variables) (*n* = 754)

**Analysis**		**Univariate**		
**Covariate**		**HR**	**95% CI**	***p* value**
Age	Cont.	1.0	0.9–1.1	0.535
AFP	Cont.	1.1	1.0–1.1	**0.009**
Albumin	Cont.	0.6	0.5–0.7	**<0.001**
Bilirubin	Cont.	1.3	1.2–1.3	**<0.001**
AST level	Cont.	1.1	1.0–1.2	**<0.001**
ALT level	Cont.	1.0	0.9–1.1	0.370
INR level	Cont.	2.2	1.6–3.1	**<0.001**
Platelet count	Cont.	1.0	0.9–1.1	0.880
Tumor number	Cont.	1.2	1.1–1.3	**<0.001**
Max. lesion size	Cont.	1.1	1.0–1.2	**<0.001**

Cont., Continuous; AFP, alpha fetoprotein; AST, aspartate aminotransferase; ALT, alanine aminotransferase. P-values in bold show significant values.

Table S8. Univariate and multivariate Cox regression analysis for all after normalizing BCA parameter to patient height in patients with available height values (continuous variables) (*n* = 522)

**Analysis**		**Univariate**			**Multivariate**		
**Covariate**		**HR**	**95% CI**	***p* value**	**HR**	**95% CI**	***p* value**
Age	Cont.	1.0	0.9 – 1.1	0.485			
AFP	Cont.	1.1	1.0 – 1.2	**0.023**	1.0	1.0 – 1.1	0.432
Albumin	Cont.	0.6	0.6 – 0.7	**<0.001**	0.7	0.6 – 0.8	**<0.001**
Bilirubin	Cont.	1.3	1.1 – 1.4	**<0.001**	1.1	1.0 – 1.2	**0.009**
AST level	Cont.	1.1	1.0 – 1.2	**0.021**	1.2	0.9 – 1.4	0.192
ALT level	Cont.	1.0	0.8 – 1.1	0.690			
INR level	Cont.	1.8	1.1 – 2.8	**0.015**	0.8	0.4 – 1.5	0.443
Platelet count	Cont.	1.0	0.9 – 1.1	0.630			
Tumor number	Cont.	1.2	1.1 – 1.3	**<0.001**	1.1	1.0 – 1.3	**0.033**
Max. lesion size	Cont.	1.4	1.2 – 1.7	**<0.001**	1.3	1.2 – 1.6	**<0.001**
SM/height^2	Cont.	0.9	0.8 – 1.0	**0.018**	0.9	0.8 – 1.0	**0.047**
TAT/height^2	Cont.	0.9	0.8 – 1.0	**0.011**	1.0	0.8 – 1.1	0.520
IMAT/height^2	Cont.	0.9	0.8 – 1.0	0.190			
SAT/height^2	Cont.	0.9	0.8 – 1.0	**0.007**	0.9	0.8 – 1.0	0.187
VAT/height^2	Cont.	0.9	0.8 – 1.0	0.110			

Cont., Continuous; AFP, alpha fetoprotein; AST, aspartate aminotransferase; ALT, alanine aminotransferase; SM, skeletal muscle volume; TAT, total adipose tissue; IMAT, intermuscular adipose tissue; VAT, visceral adipose tissue; SAT, subcutaneous adipose tissue. *Volumes normalized to squared patient height. P-values in bold show significant values.

Table S9. Univariate Cox regression analysis for BCA parameters in all patients (continuous variables) stratified by sex

**Sex**		**Male (n=622)**			**Female (n=132)**		
**Covariate**		**HR**	**95% CI**	***p* value**	**HR**	**95% CI**	***p* value**
SM normalized	Cont.	0.9	0.8 – 1.0	**0.004**	0.8	0.6 – 1.0	**0.032**
TAT normalized	Cont.	0.9	0.8 – 1.0	**0.027**	0.9	0.7 – 1.1	0.213
IMAT normalized	Cont.	1.1	0.9 – 1.1	0.499	0.9	0.7 – 1.1	0.338
SAT normalized	Cont.	0.9	0.8 – 1.0	**0.023**	0.9	0.7 – 1.0	0.125
VAT normalized	Cont.	0.9	0.8 – 1.0	0.089	1.0	0.7 – 1.3	0.876
SM/Bone	Cont.	0.8	0.8 – 0.9	**<0.001**	0.9	0.8 – 1.0	**0.042**
TAT/Bone	Cont.	0.9	0.8 – 1.0	**0.029**	0.9	0.8 – 1.0	0.175
IMAT/Bone	Cont.	1.0	0.9 – 1.1	0.449	0.9	0.8 – 1.1	0.386
SAT/Bone	Cont.	0.9	0.8 – 1.0	**0.022**	0.9	0.8 – 1.1	0.280
VAT/Bone	Cont.	0.9	0.8 – 1.0	0.104	1.0	0.8 – 1.4	0.784
Sarcopenia marker	Cont.	0.9	0.8 – 1.0	**0.011**	0.9	0.8 – 1.0	0.124

SM, skeletal muscle volume; TAT, total adipose tissue; IMAT, intermuscular adipose tissue; VAT, visceral adipose tissue; SAT, subcutaneous adipose tissue. P-values in bold show significant values.

Table S10. Univariate Cox regression analysis for BCA parameters in all patients (continuous variables) stratified by BCLC stages (A, B, C)

**BCLC stage A (n=219)**				
**Covariate**		**HR**	**95% CI**	***p* value**
SM normalized	Cont.	0.9	0.8 – 1.0	0.087
TAT normalized	Cont.	1.1	0.9 – 1.3	0.434
IMAT normalized	Cont.	1.1	1.0 – 1.3	0.155
SAT normalized	Cont.	1.0	0.9 – 1.2	0.652
VAT normalized	Cont.	1.1	0.9 – 1.2	0.465
SM/Bone	Cont.	0.9	0.8 – 1.1	**0.043**
TAT/Bone	Cont.	1.0	0.9 – 1.2	0.599
IMAT/Bone	Cont.	1.1	0.9 – 1.3	0.209
SAT/Bone	Cont.	1.1	0.9 – 1.2	0.782
VAT/Bone	Cont.	1.0	0.9 – 1.2	0.644
Sarcopenia marker	Cont.	0.9	0.7 – 1.0	**0.041**
**BCLC Stage B (n=388)**				
**Covariate**		**HR**	**95% CI**	***p* value**
SM normalized	Cont.	0.9	0.8 – 1.0	**0.033**
TAT normalized	Cont.	0.8	0.7 – 0.9	**0.001**

IMAT normalized	Cont.	0.9	0.8 – 1.0	**0.032**
SAT normalized	Cont.	0.8	0.7 – 1.0	**0.008**
VAT normalized	Cont.	0.8	0.7 – 1.0	**0.007**
SM/Bone	Cont.	0.9	0.8 – 1.0	**0.027**
TAT/Bone	Cont.	0.8	0.7 – 1.0	**0.005**
IMAT/Bone	Cont.	0.9	0.8 – 1.0	**0.032**
SAT/Bone	Cont.	0.9	0.8 – 1.0	**0.021**
VAT/Bone	Cont.	0.9	0.8 – 1.0	**0.015**
Sarcopenia marker	Cont.	1.0	0.9 – 1.1	0.857
**BCLC Stage C (n=137)**				
**Covariate**		**HR**	**95% CI**	***p* value**
SM normalized	Cont.	0.8	0.7 – 1.0	**0.041**
TAT normalized	Cont.	0.9	0.7 – 1.1	0.155
IMAT normalized	Cont.	0.9	0.8 – 1.2	0.566
SAT normalized	Cont.	0.9	0.7 – 1.1	0.213
VAT normalized	Cont.	0.9	0.7 – 1.1	0.253
SM/Bone	Cont.	0.8	0.7 – 1.0	**0.039**

TAT/Bone	Cont.	0.9	0.7 – 1.1	0.168
IMAT/Bone	Cont.	0.9	0.7 – 1.2	0.540
SAT/Bone	Cont.	0.9	0.7 – 1.1	0.249
VAT/Bone	Cont.	0.9	0.8 – 1.1	0.285
Sarcopenia marker	Cont.	0.9	0.7 – 1.0	0.184

SM, skeletal muscle volume; TAT, total adipose tissue; IMAT, intermuscular adipose tissue; VAT, visceral adipose tissue; SAT, subcutaneous adipose tissue. P-values in bold show significant values.

Fig. S1. Relation between SM and OS with cubic regression spline (knots SM quantiles)

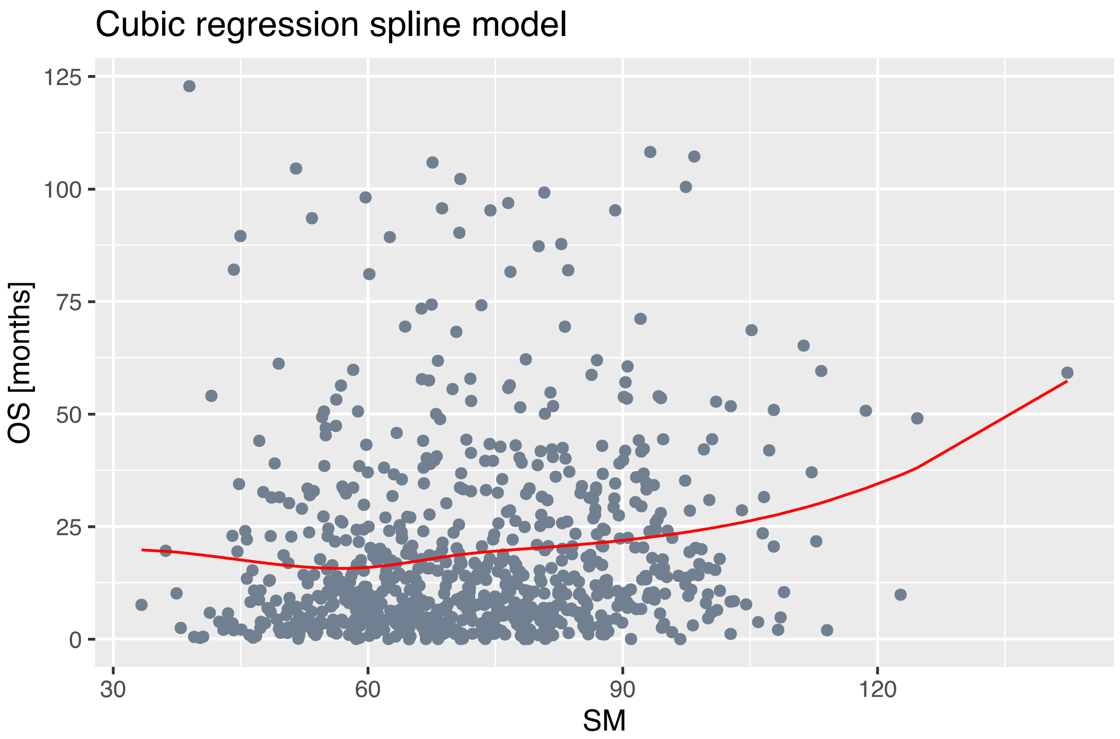

Fig. S2. Kaplan-Meier curves for the overall survival of all included patients, stratified according to previously published cut-off values for SM/Bone (Log-rank p=0.014) (A) and Sarcopenia Marker (Log-rank p<0.001) (B). SM, skeletal muscle volume.

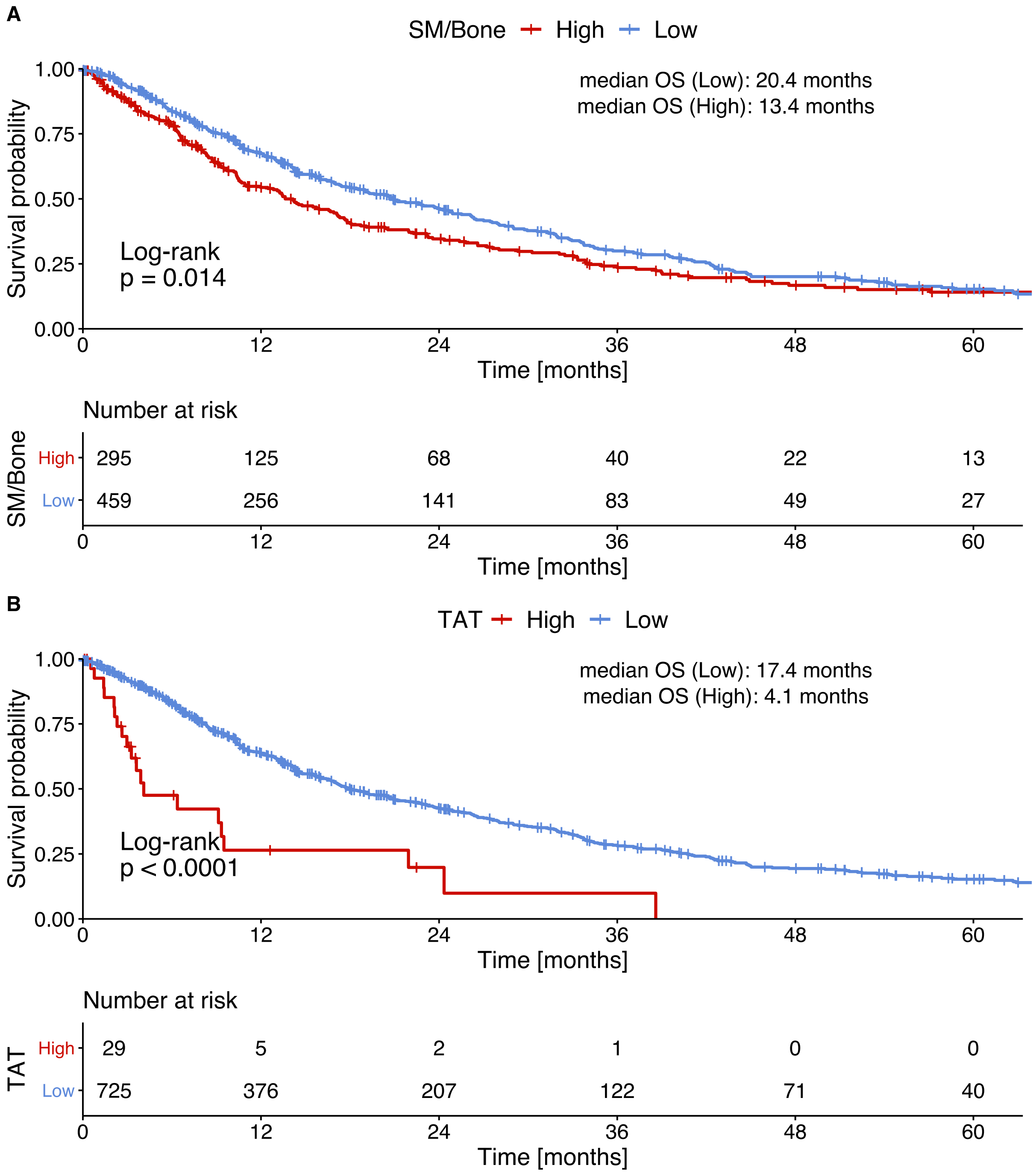

Supplement: Multimedia component 4 [file mmc4.pdf]
